# Solving Constraint Satisfaction Problems with Networks of Spiking Neurons

**DOI:** 10.3389/fnins.2016.00118

**Published:** 2016-03-30

**Authors:** Zeno Jonke, Stefan Habenschuss, Wolfgang Maass

**Affiliations:** Faculty of Computer Science and Biomedical Engineering, Institute for Theoretical Computer Science, Graz University of TechnologyGraz, Austria

**Keywords:** spiking neural networks, noise as a resource, benchmark tasks, NP-complete problems, neural sampling, neuromorphic hardware, advantage of spike-based computing, Boltzmann machine

## Abstract

Network of neurons in the brain apply—unlike processors in our current generation of computer hardware—an event-based processing strategy, where short pulses (spikes) are emitted sparsely by neurons to signal the occurrence of an event at a particular point in time. Such spike-based computations promise to be substantially more power-efficient than traditional clocked processing schemes. However, it turns out to be surprisingly difficult to design networks of spiking neurons that can solve difficult computational problems on the level of single spikes, rather than rates of spikes. We present here a new method for designing networks of spiking neurons via an energy function. Furthermore, we show how the energy function of a network of stochastically firing neurons can be shaped in a transparent manner by composing the networks of simple stereotypical network motifs. We show that this design approach enables networks of spiking neurons to produce approximate solutions to difficult (NP-hard) constraint satisfaction problems from the domains of planning/optimization and verification/logical inference. The resulting networks employ noise as a computational resource. Nevertheless, the timing of spikes plays an essential role in their computations. Furthermore, networks of spiking neurons carry out for the Traveling Salesman Problem a more efficient stochastic search for good solutions compared with stochastic artificial neural networks (Boltzmann machines) and Gibbs sampling.

## 1. Introduction

The number of neurons in the brain lies in the same range as the number of transistors in a supercomputer. But whereas the brain consumes less than 30 Watts, a supercomputer consumes as much energy as a major part of a city. Power consumption has not only become a bottleneck for supercomputers, but also for many applications of computing hardware, including the design of intelligent mobile devices. One strategy for designing substantially more power-efficient computing hardware is to port aspects of computations in networks of neurons in the brain into dedicated hardware.

The organization of computations in neural networks of the brain is apparently quite different from the organization of computations in current digital computing hardware. We propose that they are event-driven, rather than clocked, and that this feature is likely to contribute to their superior energy efficiency. The underlying events are spikes that are emitted by biological neurons at irregular intervals (Maass, 2015). One prominent open question is how the relative timing of these spikes (events) could be used for computational purposes. This question becomes especially non-trivial if one assumes that there is noise in the system that hinders straightforward deterministic approaches for simulating arbitrary Turing machines via delay-coding by spiking neurons (Maass, 1996). We present in this article a method for exploiting relative timing of spikes for computational purposes that is compatible with noise, and in fact requires noise. Rather than attempting to design networks of spiking neurons on the basis of specific neural codes and computational operations on such neural codes, we propose to focus instead on the probability distribution and dynamics of network states in a high noise regime. These network states record which neurons fire within some small time window, like in Berkes et al. (2011) and Habenschuss et al. (2013).

There exist numerous sources of noise in biological neurons and synapses (Faisal et al., 2008; Branco and Staras, 2009). A hardware emulation of stochastically firing neurons has to employ efficient methods for generating random numbers in hardware. We refer to Tetzlaff et al. (2012), Yang et al. (2014) and Al-Shedivat et al. (2015) and the emergent field of stochastic electronics (Hamilton et al., 2014) for the current state of the art in the design of efficient generators of random numbers in hardware.

We introduce new principles for the design of networks of spiking neurons for solving constraint satisfaction problems. We will illustrate these principles in applications to two well known NP-hard benchmark tasks from the domains of planning/optimization and verification/logical inference: the Traveling Salesman Problem (TSP), and satisfiability of Boolean formulas (3-SAT). The extensive literature on these two problems is reviewed in the Section 3. To the best of our knowledge there exists just one publication (Malaka and Buck, 2000) where a deterministic network of spiking neurons was applied to an instance of TSP with 8 cities. We are not aware of previous applications of networks of stochastic spiking neurons to TSP, or of any application of networks of spiking neurons to satisfiability problem.

Applications of non-spiking neural networks to solving constraint satisfaction problems had been pioneered by Hopfield and Tank (1986). Aarts and Korst (1989) give a review of applications of stochastic non-spiking neural networks (Boltzmann machines) to solving constraint satisfaction problem. These methods were based on the use of energy functions. We show how similar methods can also be used for solving constraint satisfaction problems with spiking neurons. It had already been shown in Buesing et al. (2011) that networks of stochastic spiking neurons with symmetric weights are closely related to Boltzmann machines. This relation to Boltzmann machines provides the basis for the methods that we will discuss. But we will also show in Section 2.4 that there exists an unexpected structural difference between stochastic search by Boltzmann machines and networks of spiking neurons, even if both types of networks have the same energy function. Furthermore, we will also show in Section 2.6 how networks of spiking neurons with asymmetric weights can be used for solving constraint satisfaction problems. The strategy that we propose is to retain a core network of “principal neurons” that are interconnected by symmetric weights. It turns out that with the help of a Modularity Principle (Theorem 1 in Section 2.2) one can control the marginal of the energy function for these principal neurons even if the network also employs auxiliary spiking neurons whose synaptic connections have asymmetric weights. The need for such auxiliary spiking neurons arises for example when one wants to implement the OR of 3-SAT instances with stochastic spiking neurons (Sections 2.6 and 4.2).

**Figure 1:**
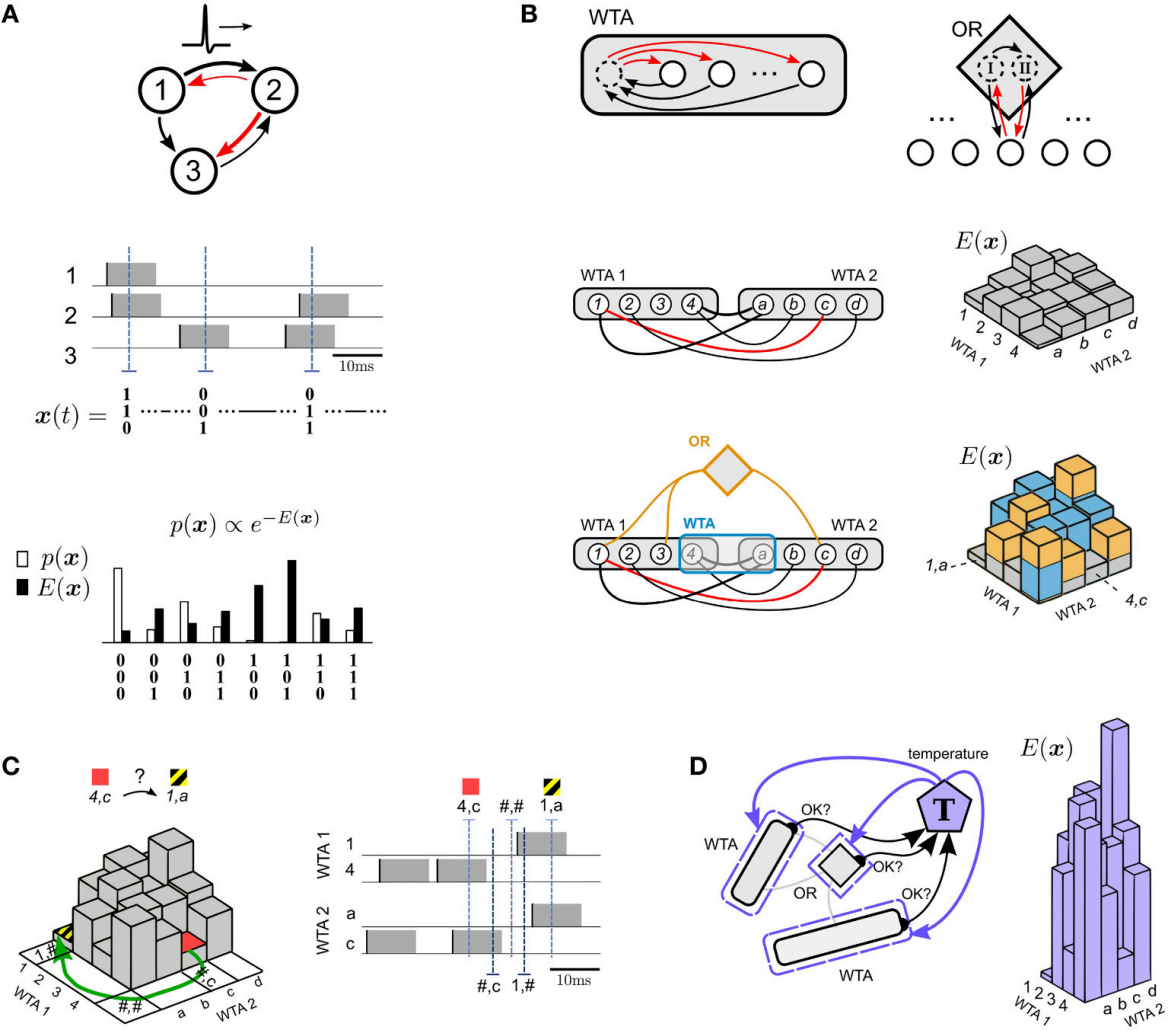
**Illustration of four principles for solving constraint satisfaction problems by spike-based neural networks with noise**. **(A)** Focus on the probability *p*(x) or energy *E*(x) of network states x, where each spike of a neuron switches a corresponding component of x to 1 for a time window of length τ that corresponds to the duration of a PSP (black/red arrows = excitatory/inhibitory connections). The network state x(*t*) can be read out at any time *t* from the network activity (3 sample time points *t* are marked by blue dashed lines in the middle plot). The energy *E*(x) of a state and its relation to the probability *p*(x) of x under the stationary distribution *p* of the network is illustrated in the bottom plot. This simple network has been chosen for illustration purposes. Its neuron numbers are not related to numbers in subsequent plots. **(B)** Modularity principle for the energy function *E*(x). The energy landscape of a subnetwork of principal neurons (white circles) can be shaped by adding circuit motifs (such as WTA, OR) with auxiliary neurons (dashed circles) that provide approximately linear contributions to *E*(x). In addition, Winner-Take-All (WTA) circuits allow evaluation of arbitrary multi-nomial problem variables, see WTA1 (for a variable ranging over {1, 2, 3, 4}) and WTA2 (for a variable ranging over {*a, b, c, d*}) in the bottom plot. Black or red lines without arrowheads denote symmetric connections. Excitatory corrections are indicated by black lines. An inhibitory connection (red arc) makes the probability of the combination of value 1 in WTA1 with value c in WTA2 particularly unlikely. This network state 1, *c* corresponds to the highest point in the corresponding energy landscape on the right. In the bottom panel two further network motifs (drawn in yellow and blue) have been added, each causing further contributions (drawn in the same colors) to the energy landscape of the simpler network in the plot above. The yellow lines of the OR motif in the bottom panel each denote 4 directed connections as indicated in the top panel (see Section 4.2.2 for details). **(C)** Spike-based dynamics supports bypassing of high-energy barriers. This example illustrates bypassing of energy barriers between low energy states 4, *c* (red) and 1, *a* (yellow/black) in the energy landscape from the bottom panel of **(B)** by moving through intermediate network states that represent undefined values of these problem variables (marked with # symbol). Note that these intermediate network states have actually high energy (not shown) because fewer terms are subtracted in the energy term (5), but nevertheless they are frequently visited as shown in Section 2.4. **(D)** Internal spike-based temperature control can drastically change the contrast of the energy landscape from **(C)** on the basis of internal information about the satisfaction of salient constraints, and thereby the progress of stochastic search. For example, an internal temperature controller *T* can drastically increase the contrast of the energy landscape from **(C)** (see illustration on the right) if all or almost all constraints are satisfied, thereby inducing the stochastic dynamics to lock into found solutions that satisfy all or most constraints.

The four principles that we are proposing for the design of networks of spiking neurons that can efficiently solve constraint satisfaction problems are formulated at the beginning of Results, and illustrated in Figure 1. Sections 2.1–2.7 discuss applications of these design principles to the TSP and 3-SAT. Further details and references to these applications can be found in the Section 4 and 5.

We would like to clarify that this article does not aim at modeling spike-based computations in biological organisms. We also would like to emphasize, that there are many other well-known methods for efficiently solving the computational tasks considered in this article, see the Section 3. The methods that we are introducing are only of interest under the premise that one wants to employ spike-based hardware for computations, e.g., because of its power-efficiency (Mead, 1990; Merolla et al., 2014).

## 2. Results

When the membrane potential of a biological neuron crosses a threshold, the neuron emits a spike, i.e., a sudden voltage increase that lasts for 1–2 ms. Spikes occur in the brain asynchronously in continuous time and are communicated to numerous other neurons via synaptic connections with different strengths (“weights”). The effect of a spike from a pre-synaptic neuron *l* on a post-synaptic neuron *k*, the so-called post-synaptic potential (PSP), can be approximated as an additive contribution to its membrane potential. It is usually modeled as being short-lived (10–20 ms) and either inhibitory or excitatory, depending on the sign of the synaptic weight *w*_*kl*_.

We model the stochastic behavior of a spiking neuron *k* at time *t* via an instantaneous firing probability (i.e., probability of emitting a spike),

(1) $$\frac{1}{\tau }\;\exp(u_{k}(t))\;,$$

that depends on the current *membrane potential u*_*k*_(*t*) of the neuron. It is defined as the weighted sum of the neuron's inputs,

(2) $$u_{k}(t)=b_{k}+\sum_{l}w_{kl}x_{l}(t)\;.$$

The additional bias term *b*_*k*_ represents the intrinsic excitability of neuron *k*. *w*_*kl*_*x*_*l*_(*t*) models the PSP at time *t* that resulted from a firing of the pre-synaptic neuron *l*. This is a standard model for capturing stochastic firing of biological neurons (Jolivet et al., 2006). We assume here for mathematical tractability rectangular PSP shapes *x*_*l*_(*t*) of length τ. The precise value of τ is not important for our results, and we set τ = 10ms. Thus, we have *x*_*l*_(*t*) = 1 if a spike was emitted by neuron *l* within (*t* − τ, *t*], and *x*_*l*_(*t*) = 0 otherwise (Figure 1A). We say then that neuron *l* is in the active state (or “on” state) during this time interval of length τ (which coincides with its refractory period). For simplicity we assume that the refractory period during which a neuron cannot spike equals τ, the length of a PSP. While the choice of the value for τ has no influence on the computational properties of the network, the choice of a different shape of PSPs causes divergences between the network dynamics and the underlying theory. More precisely, one loses precise knowledge of the stationary distribution of the network. Empirical analyses of the effects of choosing other shapes for PSPs for a somewhat different computational context can be found in Pecevski et al. (2011).

The design strategy that we are proposing for solving constraint satisfaction problems by stochastically firing neurons can be summarized by 4 principles, whose application to concrete tasks will be demonstrated in the subsequent sections. The stochastic dynamics of recurrently connected networks of spiking neurons with noise can be interpreted as search for low energy network states (principle 1, Figure 1A). This energy landscape can be shaped for concrete computational tasks in a modular fashion by composing the network from simple stereotypical network motifs (principle 2, Figure 1B). Barriers in the energy landscape can be overcome more easily if the network is forced to also assume states where none or several neurons in a WTA motif fire, corresponding to an intermittently undefined value of the corresponding multinomial variable (marked by #). This effect turns out to occur particularly often in spike-based stochastic networks (see Section 2.4) (principle 3, Figure 1C). Finally, networks of spiking neurons can internally rescale their energy landscape in order to lock into desirable solutions (principle 4, Figure 1D).

### 2.1. Network states, stationary distributions, and energy functions

We propose to focus on the temporal evolution and statistics of spike-based network states (principle 1), rather than on spikes of individual neurons, or rates, or population averages. The *network state* of a network of *N* neurons at time *t* is defined here as x(*t*) = (*x*_1_(*t*), *x*_2_(*t*), …, *x*_*N*_(*t*)) (Figure 1A, middle), where *x*_*k*_(*t*) = 1 indicates that neuron *k* has fired (i.e., emitted a spike) during the time interval (*t* − τ, *t*] corresponding to the duration of a PSP. Else *x*_*k*_(*t*) = 0. If there is a sufficient amount of noise in the network, caused for example by the stochastic firing of neurons according to Equation (1), the distribution of these network states converges exponentially fast from any initial state to a unique stationary (equilibrium) distribution *p*(x) of network states (Habenschuss et al., 2013). This stationary distribution *p*(x) can be viewed as a concise representation of the statistical fine-structure of network activity at equilibrium. In line with the related non-spiking neural network approach of Hopfield and Tank (1986) we will use in the following an alternative representation of *p*(x), namely the energy function *E*(x) = −log *p*(x) + *C*, where *C* denotes an arbitrary constant (Figure 1A bottom), so that low energy states occur with high probability at equilibrium. If exactly one neuron of a WTA circuit with *K* competing neurons fires (see Figure 1B for the case *K* = 4), one can interpret the current state of this WTA circuit as the current value of a multinomial variable with *K* values. But one should keep in mind that the resulting notion of network state x with multinomial rather than binary components only provides a heuristic description of the true network state, which is always a binary vector (as defined above). The difference becomes apparent when for a moment none or more than 1 neuron of a WTA circuit is in the on-state. In this case the binary network state is still well-defined, but not the heuristic version in terms of multinomial variables. This case is considered in Figure 1C, and turns out to be important for Section 2.4.

The *neural sampling* theory (Buesing et al., 2011) implies that the stationary distribution of a network with neuron model given by Equation (1) and (2), and symmetric weights *w*_*kl*_ = *w*_*lk*_ is a Boltzmann distribution with energy function

(3) $$E_{N}(x)=-\sum_{k=1}^{N}b_{k}x_{k}-\frac{1}{2}\sum_{k=1}^{N}\sum_{l=1}^{N}x_{k}x_{l}w_{kl}\;.$$

This energy function has at most second-order terms. This suffices for solving the TSP. But many other constraint satisfaction problems (such as 3-SAT, see below) require the modeling of higher-order dependencies among problem variables. To introduce higher-order dependencies among a given set of *principal* neurons x, one needs to introduce additional *auxiliary* neurons ξ to emulate the desired higher-order terms. Two basic approaches can be considered. In the first approach one assumes that synaptic connections between the principal neurons x and the auxiliary neurons ξ, as well as the connections within each group are symmetric, i.e., bidirectional, with equal weights in both directions. In such case, the energy function *E*_N_(x, ξ) of the joint distribution over principal neurons and auxiliary neurons can be described with at most second order terms. But the marginal energy function for just the principal neurons,

(4) $$E_{N}(x)=\log\sum_{\xi }\exp(E_{N}(x,\xi ))\;\;,$$

with auxiliary neurons marginalized out, will then contain in general also higher-order terms. By clever use of symmetrically connected auxiliary neurons one can thereby introduce arbitrary higher-order dependencies among principal neurons. In practice, however, this “symmetric” approach has been found to substantially slow down convergence to the stationary distribution (Pecevski et al., 2011), due to large energy barriers introduced in the energy landscape when one introduces auxiliary variables through deterministic definitions.

The alternative approach, which is pursued in the following for 3-SAT, is to maintain symmetric connections among principal network neurons, but to abandon the constraint that connections between principal and auxiliary neurons, as well as connections among auxiliary neurons, have to be symmetric. Furthermore, auxiliary variables or neurons are related by stochastic (rather than deterministic) relationships to principal neurons, thereby supporting fast convergence to the stationary distribution. The theoretical basis for constructing appropriate auxiliary network motifs is provided by the neural computability condition (NCC) of Buesing et al. (2011). The NCC states that it suffices for a neural emulation of an arbitrary probability distribution *p*(x) over binary vectors *x* that there exists for each binary component *x*_*k*_ of *x* some neuron *k* with membrane potential

(5) $$u_{k}(t)=\log\frac{p(x_{k}=1|x_{\setminus k}(t))}{p(x_{k}=0|x_{\setminus k}(t))}\;,$$

where x_\*k*_(*t*) denotes the state of all neurons except neuron *k*. For a second-order Boltzmann distribution, evaluating the right-hand side gives the simple linear membrane potential in Equation (2). For more complex distributions, additional higher-order terms appear.

### 2.2. Modularity of the energy function

The shaping of the energy function of a network of spiking neurons with asymmetric connections or weights can be simplified through a modularity principle (see Figure 1B, principle 2). It allows us to understand the energy function *E*(x) of a large class of networks of spiking neurons in terms of underlying generic network motifs.

As introduced above, we distinguish between principal neurons and auxiliary neurons: Principal neurons constitute the interface between network and computational task. For example, principal neurons can directly represent the variables of a computational problem, such as the truth values in a logical inference problem (**Figure 4**). The state x(*t*) of the *principal network* (i.e., the principal neurons) reflects at any moment *t* an assignment of values to the problem variables. Auxiliary neurons, on the other hand, appear in specialized network motifs that modulate the energy function of the principal network. More specifically, the purpose of auxiliary neurons is to implement higher-order dependencies among problem variables. The starting point for constructing appropriate auxiliary circuits is the NCC from Equation (5) rewritten in terms of energies from Equation (3),

(6) $$u_{k}(t)=E\left(x_{k}=0,\;x_{\setminus k}(t)\right)-E\left(x_{k}=1,\;x_{\setminus k}(t)\right)\;.$$

This sufficient condition supports the following strategy for engaging auxiliary neurons that are not subject to the constraint given by Equation (6), in order to shape the energy function *E*(x) of the principal network in desirable ways: Suppose that a set of auxiliary circuits I is added (and connected with symmetric or asymmetric connections) to a principal network with energy function given by Equation (3). Due to linearity of membrane integration according to Equation (2) the membrane potential of a principal neuron *k* in the presence of such auxiliary circuits can be written as,

(7) $$u_{k}(t)=b_{k}+\sum_{l=1}^{N}w_{kl}x_{l}(t)+\sum_{i\in ℐ}u_{k,i}(t)\;,$$

where the instantaneous impact of auxiliary circuit *C*_*i*_ on the membrane potential of principal neuron *k* is denoted by *u*_*k*, *i*_(*t*). In the presence of such auxiliary circuits there is no known way to predict the resulting stationary distribution *p*(x) over principal network states (or equivalently *E*(x)) *in general*. However, the following Theorem implies that under some conditions each auxiliary motif makes a transparent *linear* contribution to the energy function *E*(x) of the principal network (see blue and yellow circuit motifs in Figure 1B bottom).

**Theorem 1 (Modularity Principle)**. *Let* N *be a network of stochastic neurons k* = 1, …, *N according to Equation (*1*) *and* (*2*), symmetric connections w*_*kl*_ = *w*_*lk*_ *(but no self-connections, i.e*., *w*_*kk*_ = 0*) and biases b*_*k*_*. In the absence of auxiliary circuits this principal network has an energy function E*_N_(x) *with first- and second-order terms as defined in Equation* (3). *Let* C = {*C*_1_, …, *C*_*L*_} *be a set of L additional auxiliary circuits which can be reciprocally connected to the principal network* N *to modulate the behavior of its neurons. Suppose that for each auxiliary circuit C*_*i*_ *there exists a function U*_*i*_(*x*) *such that at any time t the following relation holds for the impact u*_*k*, *i*_(*t*) *of circuit C*_*i*_ *on the membrane potential u*_*k*_(*t*) *of any neuron k in* N*:*

(8) $$u_{k,i}(t)=U_{i}\left(x_{k}=0,x_{\setminus k}(t)\right)-U_{i}\left(x_{k}=1,x_{\setminus k}(t)\right)\;,$$

*The relation in Equation* (8) *is assumed to hold for each auxiliary circuit C*_*i*_ *regardless of the presence or absence of other auxiliary circuits*.

*Then the modulated energy function E*_N, I_(x) *of the network in the presence of some arbitrary subset* I⊆{1, …, L} *of auxiliary circuits can be written as a linear combination:*

(9) $$E_{N,ℐ}(x)=E_{N}(x)+\sum_{i\in ℐ}U_{i}(x)\;\;\;.$$

Examples for network motifs that impose computationally useful higher order constraints in such modular fashion are the WTA and OR motif (Figure 1B). The WTA motif (see Supplementary Material Section 3.2) is closely related to ubiquitous motifs of cortical microcircuits (Douglas and Martin, 2004). It increases the energy of all network states where not exactly one of the *K* principal neurons to which it is applied is active (this can be realized through an auxiliary neuron mediating lateral inhibition among principal neurons). It may be used, for example, to enforce that these *K* principal neurons represent a discrete problem variable with *K* possible values. The OR-motif, which can be applied to any set of principal neurons, enforces that most of the time at least one of these principal cells is active. It can be implemented through two auxiliary neurons *I* and *II* which are reciprocally connected to these principal neurons (as illustrated in Figure 1B for two principal neurons). Neuron *I* exciteps them, and neuron *II* curtails this effect through subsequent inhibition as soon as one of them fires, see Supplementary Material Section 3.3.

### 2.3. Application to the traveling salesman problem

We first demonstrate the computational capabilities that spiking networks gain through these principles in an application to the TSP, a well-known difficult (in fact NP-hard) benchmark optimization problem (see Methods for previous results and references). TSP is a planning problem where given analog parameters *c*_*ij*_ represent the cost to move directly from a node (“city”) *i* in a graph to node *j*. The goal is to find a tour that visits all *N* nodes in the graph exactly once, with minimal total cost.

A network of spiking neurons that produces approximate solutions for the TSP can be constructed as follows: One dedicates one WTA circuit or module *X*_*s*_ to each time step *s* of the tour. This WTA module consists of *N* neurons, one for each of the *N* cities that could possibly be visited at step s. Interconnections with strong negative weights between the *N* neurons in each WTA module ensure that usually at most one of its *N* neuron fires at any moment in time. The index *i* of the neuron in WTA module *X*_*s*_ that has most recently fired can be interpreted as a current proposition to visit city *i* at step *s* of the tour (using the same translation from spikes to bits as in Figure 1A. Thus, by recording for each WTA module which neuron has fired most recently, one can decode at any time *t* the firing activity of the whole network as a proposed TSP solution.

**Figure 2:**
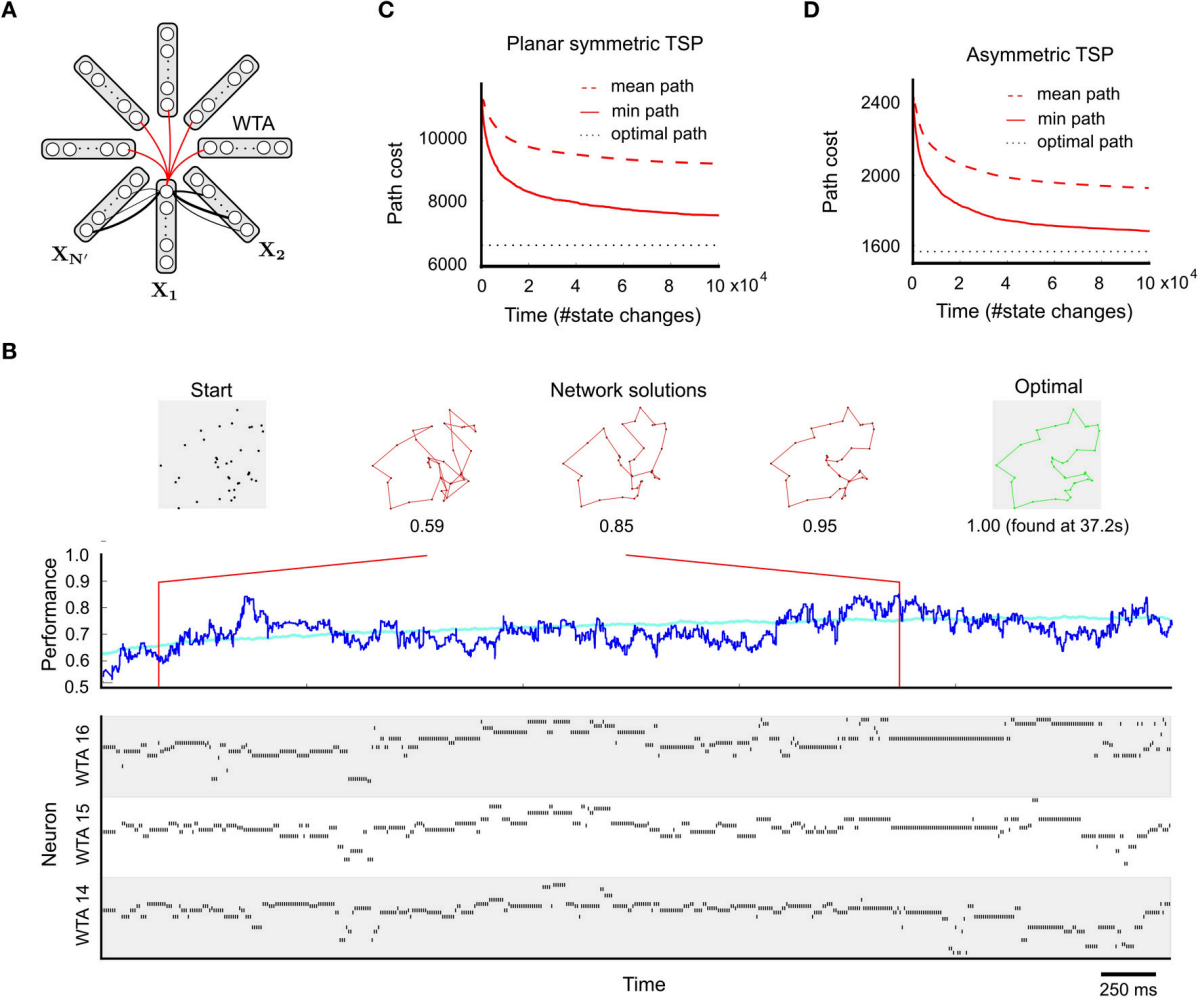
**Application to the TSP**. **(A)** Network design, with one WTA circuit *X*_*n*_ for each of the *N*′ = *N* + *N*_*resting*_ steps of a tour through *N* cities (see main text). The synaptic weights between two steps are chosen to reflect movement costs between each pair of cities. The constraint that each city must be visited exactly once is enforced by inhibitory connections among neurons coding for the same city at different time steps. **(B)** Example application to a planar TSP instance with *N* = 38. Top: 38-city problem on the left, tours generated by the network in the middle, and the optimal solution on the right. Bottom: spike trains of neurons in selected WTA circuits during the first few seconds of a typical run. Middle: network performance over time (dark blue: single trial, cyan: average over 100 trials). The network quickly generates good approximate solutions to the TSP problem. **(C)** Mean and minimum path length (cost) of solutions generated within a given number of state changes (average over 100 runs; a state change happens whenever a neuron spikes or its PSP ends τ time units later), for the planar 38-city problem of **(B)**. **(D)** Performance for an asymmetric TSP problem (N = 39). Also here the network produces relatively fast good approximations (note that path costs in **(C,D)** are not comparable). Panels **A** and **B** are reprinted from Maass (2015) with kind permission from IEEE.

The black links between adjacent WTA modules in Figure 2A (that select cities for adjacent steps of the tour) denote excitatory synaptic connections, whose strengths (weights) encode the cost matrix for the *N* cities. These weights are larger for synaptic connections between neurons that encode cities *i* and *j* for which a transition has lower cost, see Equation 15 for the precise definition. A larger weight increases the probability that if one of these two neuron fires, the other one also fires, thereby suggesting to go from city *i* directly to city *j* (or vice versa). Note that the costs of the two directions are encoded by two different synaptic weights that can be chosen independently. Strongly negative weights on synaptic connections (red arcs in Figure 2A) between neurons with the same index *i* in different WTA modules reduce the probability that a tour is proposed where city *i* is visited repeatedly. Figures 2B–D show that the network finds some reasonable solution quite fast, and keeps finding better solutions when it has more time to search.

A few (*N*_*resting*_) extra steps (i.e., WTA circuits) are introduced to create an energy landscape with additional paths to low energy states. Cases where the costs are symmetric (*c*_*ij*_ = *c*_*ji*_), or even represent Euclidean distances between nodes in 2D space, are easier to visualize (Figure 2B, top) but also computationally easier. Nevertheless, also general TSP problems with asymmetric costs can be solved approximately by spike-based circuits with synaptic weights (Figure 2D). This is possible because of the described coding method where different synaptic connections encode the cost of moving from city *i* to city *j* and the cost of moving from city *j* to city *i*.

### 2.4. Advantage of spike-based stochastic search

While the design of network motifs benefits already from the freedom to make synaptic connections asymmetric (consider e.g., in- and out-going connections of the auxiliary WTA neuron that implements lateral inhibition), our third principle proposes to exploit an additional generic asymmetry of spike-based computation. This concerns an asymmetry in the dynamics of spiking neurons, and has no relationship to asymmetry in synaptic weights. A spike which changes the state of a neuron *k* to its active state, occurs randomly in time according to Equation (1). But its transition back to the inactive state occurs deterministically τ time units later. As a result, it may occur for brief moments that all *K* principal neurons of a WTA motif are inactive, rendering an associated *K*-valued problem variable to be intermittently undefined. Most of the time this has no lasting effect because the previous state is quickly restored. But when the transition to an undefined variable state occurs in several WTA circuits at approximately the same time, the network state can bypass high-energy barriers and explore radically different state configurations (Figure 1C). Our theoretical analysis implies that this effect enhances exploration in spike-based networks, compared with Boltzmann machines (Gibbs sampling). The TSP is a suitable study case for such comparison, because we can compare the dynamics of a spiking network for the TSP with that of a Boltzmann machine which has exactly the same stationary distribution (i.e., energy function).

#### 2.4.1. Specific properties of spike-based stochastic dynamics

Consider a Boltzmann machine or Gibbs sampler (Brooks et al., 2010) (operating in continuous time to allow for a fair comparison; for details see Supplementary Material Section 4.4) that samples from the same distribution *p*(x) as a given spiking network. Such non-spiking Gibbs sampler has a symmetric transition dynamics: it activates units proportional to the sigmoid function σ(*u*) = (1 + *exp*(−*u*))^−1^, while off-transitions occur at a rate proportional to σ(−*u*). Neural sampling in a stochastic spiking network, on the other hand, triggers on-transitions proportional to *exp*(*u*), while off-transitions occur at a constant “rate.” In neural sampling, the mean transition times *m*_*on*_ from the last *on*→*off* to the next *off*→*on* transition and its dual *m*_*off*_ in a neuron with membrane potential *u* are given by:

(10) $$m_{\text{on}}(u)=\tau \cdot \exp(-u)\;,$$

(11) $$m_{\text{off}}(u)=\tau \;.$$

**Figure 3:**
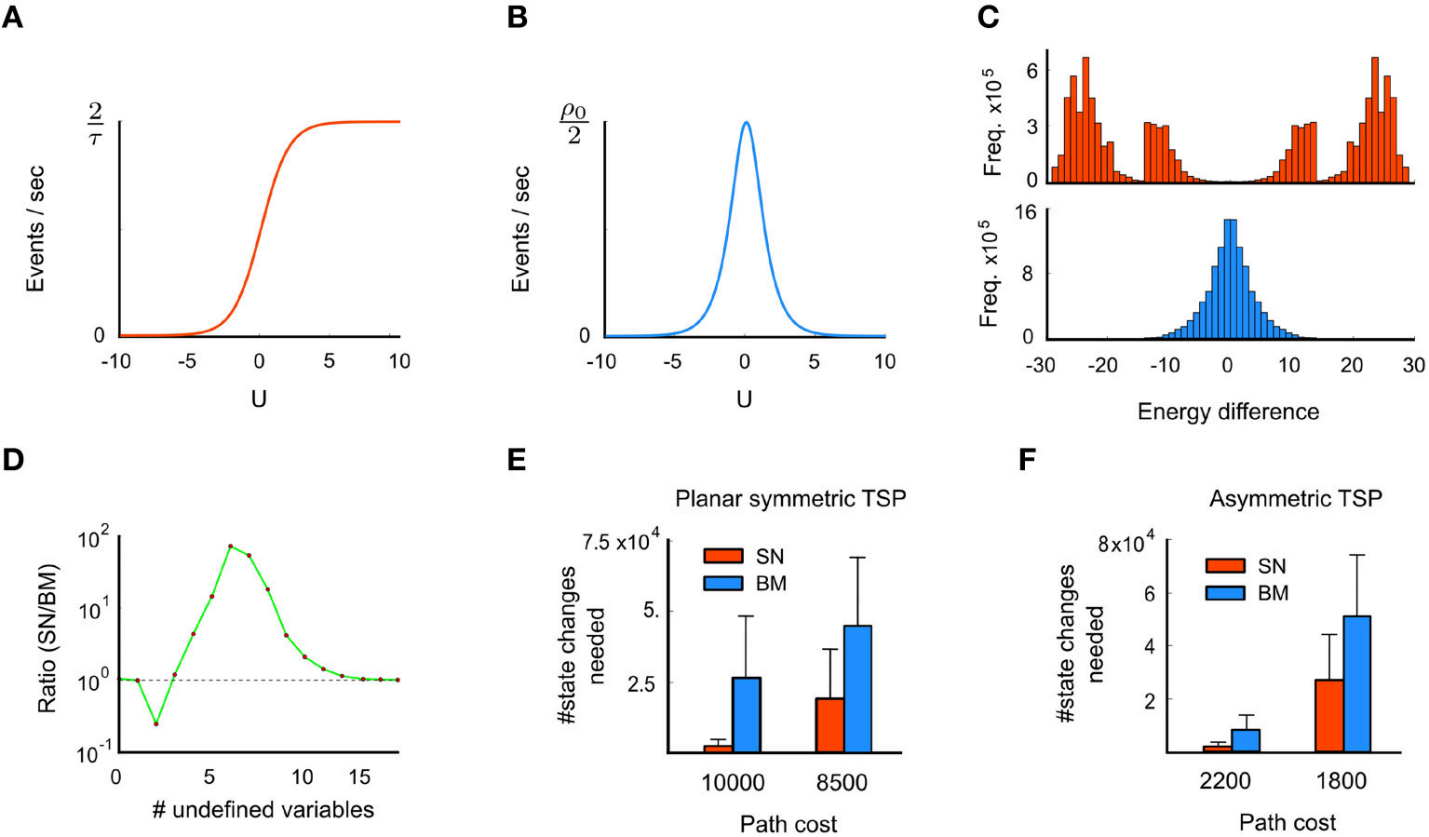
**Advantage of spike-based stochastic search (third principle)**. **(A)** The event rate (counting both on and off events) of a spiking neuron is low for small membrane potentials *u*, and saturates for high *u*. **(B)** In contrast, the event rate of a Boltzmann unit is sharply peaked around *u* = 0. **(C)** Histogram of energy jumps during the stochastic dynamics of a spiking network (SN) at the top and a Boltzmann machine (BN) with the same energy function at the bottom. Energy jumps are due to state changes, caused by neural on- or off-transitions in the network. **(D)** Comparison between SN and BM regarding the frequency of state transitions that leave one or several problem variables undefined. Shown is the ratio SN/BM for the symmetric TSP problem considered in Figure 2. Transitions into states with 5 or more undefined variables occur with higher frequency in the SN. **(E)** Comparison of average computation times for 100 runs with standard deviations (in terms of the number of state changes of the network) until a tour with a given cost is found for the problem instance from Figure 2B, for spike-based networks and corresponding Boltzmann machines. **(F)** Same for the 39 city problem with asymmetric costs considered in Figure 2D. These results indicate specific advantages of spike-based stochastic search for these two problem instances. Panels **C** and **E** are reprinted from Maass (2015) with kind permission from IEEE.

On average, an off-and-on (or on-and-off) transition sequence takes *m*_*on*_(*u*) +*m*_*off*_(*u*) time units. Thus, the average event rate *R*(*u*) at which a spiking neuron with membrane potential *u* changes its state is given by (Figure 3A),

(12) $$R(u)=\frac{2}{m_{on}(u)+m_{off}(u)}=\frac{2}{\tau }\sigma (u)\;\;\;\;\;.$$

In contrast, the average event rate *R*^*sym*^(*u*) in a Gibbs sampler at membrane potential *u* is given by (Figure 3B),

(13) $$R^{sym}(u)=\frac{2\rho_{0}}{2+exp(u)+exp(-u)}\;\;\;\;\;,$$

where ρ_0_ is a positive constant which controls the overall speed of sampling in the continuous-time Gibbs sampler, with the default choice being ρ_0_ = 1. Clearly, although asymmetric spiked-based and symmetric Gibbs sampling both sample from the same distribution *p*(x) of states of the principal network, the frequency of state transitions at different levels of the membrane potential *u* differs quite drastically between the two samplers. Concretely, the following *u*-dependent factor *F*(*u*) relates the two systems:

(14) $$F(u)=\frac{R(u)}{R^{sym}(u)}=\frac{1}{\underset{\text{const.}}{\underset{︸}{\tau \rho_{0}}}}\cdot \left(1+\exp(u)\right)\;.$$

Similar to τ in the spike-based system, ρ_0_ can be understood as a global time scale parameter which has no bearing on the fine-scale dynamics of the system. The remaining factor reveals a specific *u*-dependence of transition times which greatly affects the temporal dynamics of sampling. Note that *F*(*u*) is strictly positive and increases monotonically with increasing membrane potential *u*. Hence, the asymmetric dynamics of spiking neurons increases specifically the *on*- and *off* -transition rates of neurons with high membrane potentials *u* (i.e., neurons with strong input and/or high biases). According to Equation (6), however, high membrane potentials *u* reflect large energy barriers in the energy landscape. Therefore, the increase of transition rates for large *u* in the spike-based system (due to the asymmetry introduced by deterministic *on*→*off* transitions) means that large energy barriers are expected to be crossed more frequently than in the symmetric system (see Figure 1C and Supplementary Material Section 4 for further details).

#### 2.4.2. Spikes support bypassing of high energy barriers

A specific computational consequence of spike-based stochastic dynamics is demonstrated in Figure 3 for the TSP problems of Figure 2: Transitions that bridge large energy differences occur significantly more frequently in the spiking network, compared to a corresponding non-spiking Boltzmann machine or Gibbs sampling (Figure 3C). In particular, transitions with energy differences beyond ± 15 are virtually absent in Gibbs sampling. This is because groups of neurons that provide strong input to each other (such that all neurons have a high membrane potential *u* > 15) are very unlikely to switch off once they have settled into a locally consistent configuration (due to low event rates at high *u*, see Figure 3B). In the spiking network, however, such transitions occur quite frequently, since neurons are “forced” to switch off after τ time units even if they receive strong input *u*, as predicted by Figure 3A. To restore a low-energy state, the neuron (or another neuron in the same WTA circuit with similarly strong input *u*) will likely fire very soon afterwards. This gives rise to the observed highly positive and negative energy jumps in the spiking network, see Supplementary Material for explanations of further details of Figure 3C. As a consequence of increased state transitions with large energy differences, intermittent transitions into states that leave many problem variables undefined are also more likely to occur in the spiking network (Figures 1C, D). Note that the concrete shape of the curves in Figures 3C,D results from the interaction of the theoretical principles of Section 2.4.1 with the specific stochastic dynamics of the networks for the chosen TSP instances.

In order to avoid misunderstandings, we would like to emphasize that such states with undefined problem variables have nothing to do with neurons being in their refractory state, because the state of a neuron that is in its refractory state is well-defined (with value 1) during that period.

Consistent with the idea that state transitions with large energy differences facilitate exploration, the spiking network needs for the chosen TSP instances significantly fewer network state changes to arrive at tours with a given desired cost than the corresponding Boltzmann machine (Figures 3E,F). Significance was evaluated through a two-sample Kolmogorov-Smirnov test, see Section 4.1.2 for details. This indicates for these problem instances an advantage of spike-based computation for stochastic search. For the case of Boltzmann machines in discrete time, which is the commonly considered version, the performance difference to spiking neural networks might be even larger.

Finally, we would like to mention that there exist other stochastic search methods (e.g., the Metropolis-Hastings algorithm) that are in general substantially more efficient than Gibbs sampling. Hence the preceding results are only of interest if one wants to execute stochastic search by a distributed network in asynchronous continuous time with little or no overhead.

### 2.5. Spike-based internal temperature control

Most methods that have been proposed for efficient search for low energy states in stochastic systems rely on an additional external mechanism that controls a scaling factor *T* (“temperature”) for the energy contrast between states, *E*_*T*_(x) = *E*(x)∕*T* (with appropriate renormalization of the stationary distribution). Typically these external controllers lower the temperature *T* according to some fixed temporal schedule, assuming that after initial exploration the state of the system is sufficiently close to a global (or good local) energy minimum. We propose (principle 4) to exploit instead that a spiking network has in many cases internal information available about the progress of the stochastic search, e.g., an estimate of the energy of the current state. Since we have the freedom to use asymmetric synaptic weights, dedicated circuit motifs can internally collect this information and activate additional circuitry that emulates an appropriate network temperature change (Figure 1D).

More concretely, in order to realize an internal temperature control mechanism for emulating temperature changes of the network energy function according to *E*_*T*_(x) = *E*(x)∕*T* in an autonomous fashion, at least three functional components are required: (a) Internally generated feedback signals from circuit motifs reporting on the quality and performance of the current tentative network solution. (b) A *temperature control* unit which integrates feedback signals and decides on an appropriate temperature *T*. (c) An implementation of the requested temperature change in each circuit motif.

Both circuits motifs, WTA and OR, can be equipped quite easily with the ability to generate internal feedback signals. The WTA constraint in a WTA circuit is met if exactly one principal neuron is active in the circuit. Hence, the summed activity of WTA neurons indicates whether the constraint is currently met. Similarly, the status of an OR constraint can be read out through an additional status neuron which is spontaneously active but deactivated whenever one of the OR neurons fires. The activity of the additional status neuron then indicates whether the OR constraint is currently violated.

Regarding the temperature control unit, one can think of various smart strategies to integrate feedback signals in order to decide on a new temperature. In the simplest case, a temperature control unit has two temperatures to choose from: one for exploration (high temperature), and one for stabilization of good solutions (low temperature). A straightforward way of selecting a temperature is to remain at a moderate to high temperature (exploration) by default, but switch temporarily to low temperature (stabilization) whenever the number of positive feedback signals exceeds some threshold, indicating that almost all (or all) constraints in the circuit are currently fulfilled.

**Figure 4:**
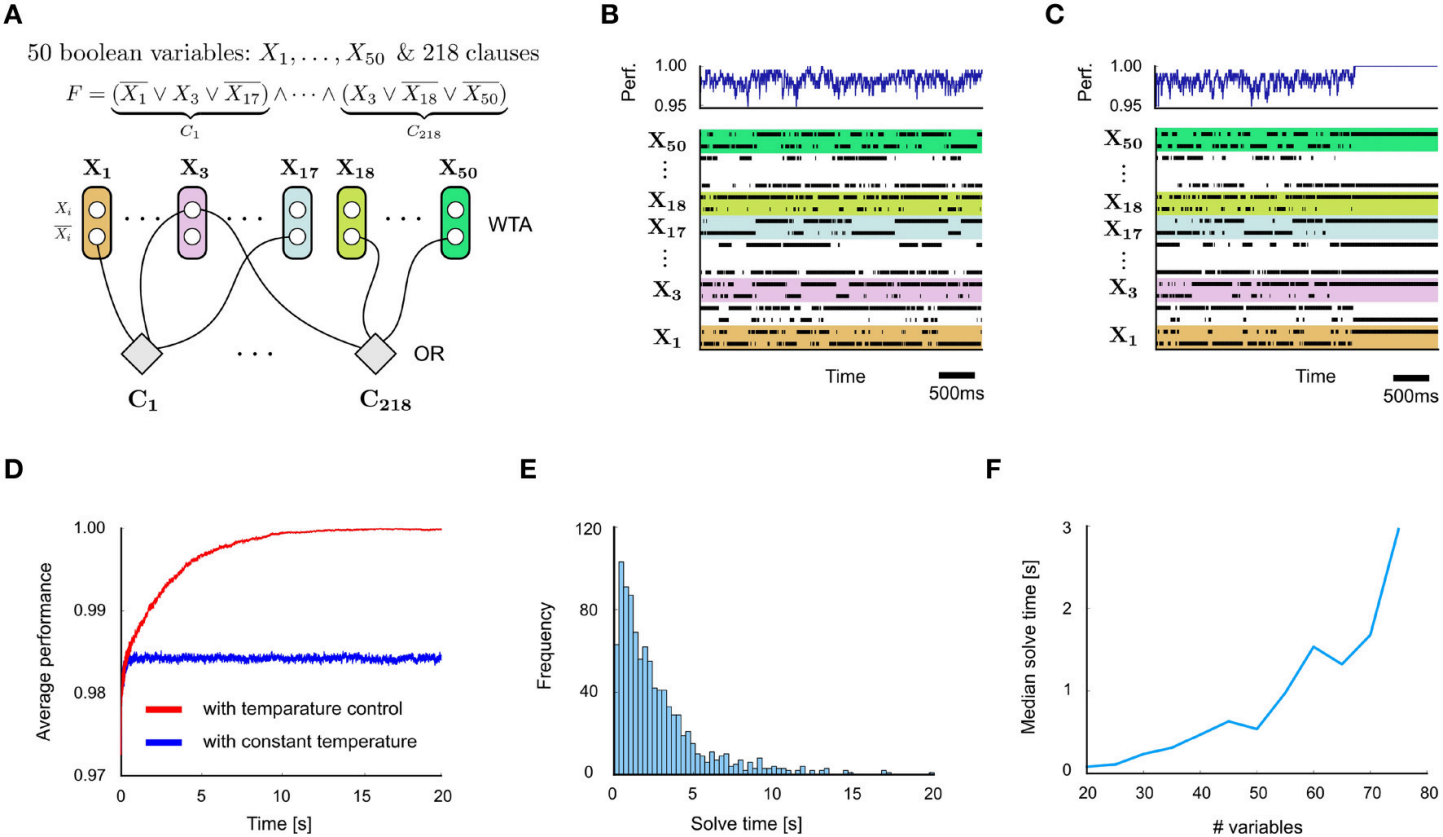
**Application to 3-SAT**. **(A)** Network design for a problem instance with 50 Boolean variables and 218 clauses. **(B)** Spiking activity of some of the neurons in the network (bottom) and quality of problem solution (% of clauses satisfied) corresponding to each network state. **(C)** Same with internal temperature control circuit motifs added. The network locks after a while into a perfect solution. **(D)** Comparison of quality of solutions represented by networks states x(*t*) in networks with and without internal temperature control (average over 100 runs). **(E)** Distribution of solve times, i.e., the time needed until a satisfying solution is found for the first time, for the Boolean formula from **(A)** (with internal temperature control). **(F)** Test of scalability of spike-based design principles to instances of 3-SAT with different numbers of variables, but a fixed clause/variable ratio of 4.3. The solve time grows quickly with the size of the problem, as expected for NP-hard problems. Nevertheless, spike-based networks are typically able to solve fairly large hard 3-SAT instances within a few seconds.

Concretely, such an internal temperature control unit can be implemented via a temperature control neuron with a low bias and connection strengths from feedback neurons in each circuit in such a manner that the neuron's firing probability reaches non-negligible values only when all (or almost all) feedback signals are active. When circuits send either positive or negative feedback signals, the connection strengths from negative feedback neurons should be negative and can be chosen in such a manner that non-negligible firing rates are achieved only if all positive feedback but none of the negative feedback signals are active. When the temperature control neuron fires it indicates that the circuit should be switched to the low temperature (stabilization) regime. We show in Figures 4C,D how this mechanisms improves the performance of a spiking network for 3-SAT: by locking into a satisfying solution for a given Boolean formula once it has been found. For details see Supplementary Material Section 5.

### 2.6. Application to the satisfiability problem (3-SAT)

We demonstrate internal temperature control in an application to 3-SAT, another well-studied benchmark task (Figure 4A). 3-SAT is the problem to decide whether a Boolean formula *F* involving *N* Boolean variables *X*_*i*_, …, *X*_*N*_ is satisfiable (or equivalently, whether its negation is not provable), for the special case where *F* is a conjunction (AND) of clauses (OR's) over 3 literals, i.e., over Boolean variables *X*_*n*_ or their negations *X*_*n*_. 3-SAT is NP-complete, i.e., there are no known methods that can efficiently solve general 3-SAT problems. We refer to the Section 3 for a sketch of the state-of-the-art and references. We are not aware of spiking neural network implementations for solving satisfiability problems.

Despite exponential worst-case complexity, many 3-SAT instances arising in practice can be solved quite efficiently by clever heuristic algorithms (Gomes et al., 2008). A class of particularly hard 3-SAT instances can be found in a subset of random 3-SAT problems. In a uniformly random 3-SAT problem with *N* Boolean variables and *M* clauses, the literals of each clause are chosen at random from a uniform distribution (over all possible 2*N* literals). The typical hardness of a random 3-SAT problem is determined to a large extent by the ratio *M*∕*N* of clauses to variables. For small ratios almost all random 3-SAT problems are satisfiable. For large ratios almost all problems are unsatisfiable. For large problems *N* ≫ 1 one observes a sharp phase transition from all-satisfiable to all-unsatisfiable at a crossover point of *M*∕*N* ≈ 4.26. For smaller *N* the transition is smoother and occurs at slightly higher ratios. Problems near the crossover point appear to be particularly hard in practice (Crawford and Auton, 1996).

For hard random instances of 3-SAT, like those considered in Figure 4 with a clauses-to-variables ratio 4.3 near the crossover point, typically only a handful of solutions in a search space of 2^N^ possible assignments of truth values to the Boolean variables exist. A spike-based stochastic circuit that searches for satisfying value assignments of a 3-SAT instance *F* can be constructed from WTA- and OR-modules in a straightforward manner (Figure 4A). Each Boolean variable *X*_*n*_ is represented by two neurons ν_*n*0_ and ν_*n*1_, so that a spike of neuron ν_*ni*_ sets the value of *X*_*n*_ to *i* for a time interval of length τ. A WTA circuit ensures that for most time points *t* this holds for exactly one of these two neurons. Otherwise *X*_*n*_ is undefined at time *t*. An OR-module for each clause of *F* increases the energy function of the network according to the number of clauses that are currently unsatisfied. An internal spike-based temperature controller can easily be added via additional modules, for details see Supplementary Material Section 5. It considerably improves the performance of the network (Figures 4C,D), while keeping the total number of neurons linear in the size of the problem instance *F*.

### 2.7. Role of precise timing of spikes

In spite of the stochasticity of the spiking network, the timing of spikes and subsequent PSPs plays an important role for their computational function. Like in a Boltzmann machine with asynchronous updates of units in continuous time, errors arise when an update of a neuron does not take into account recent updates of other neurons. In particular, in spike-based WTA circuits only 1 out of *N* neurons should be active in order to transmit correct information to other neurons. Transmission delays increase the probability that multiple neurons in a WTA circuit become simultaneously active. This may cause ambiguous messages to other parts of the network.

**Figure 5:**
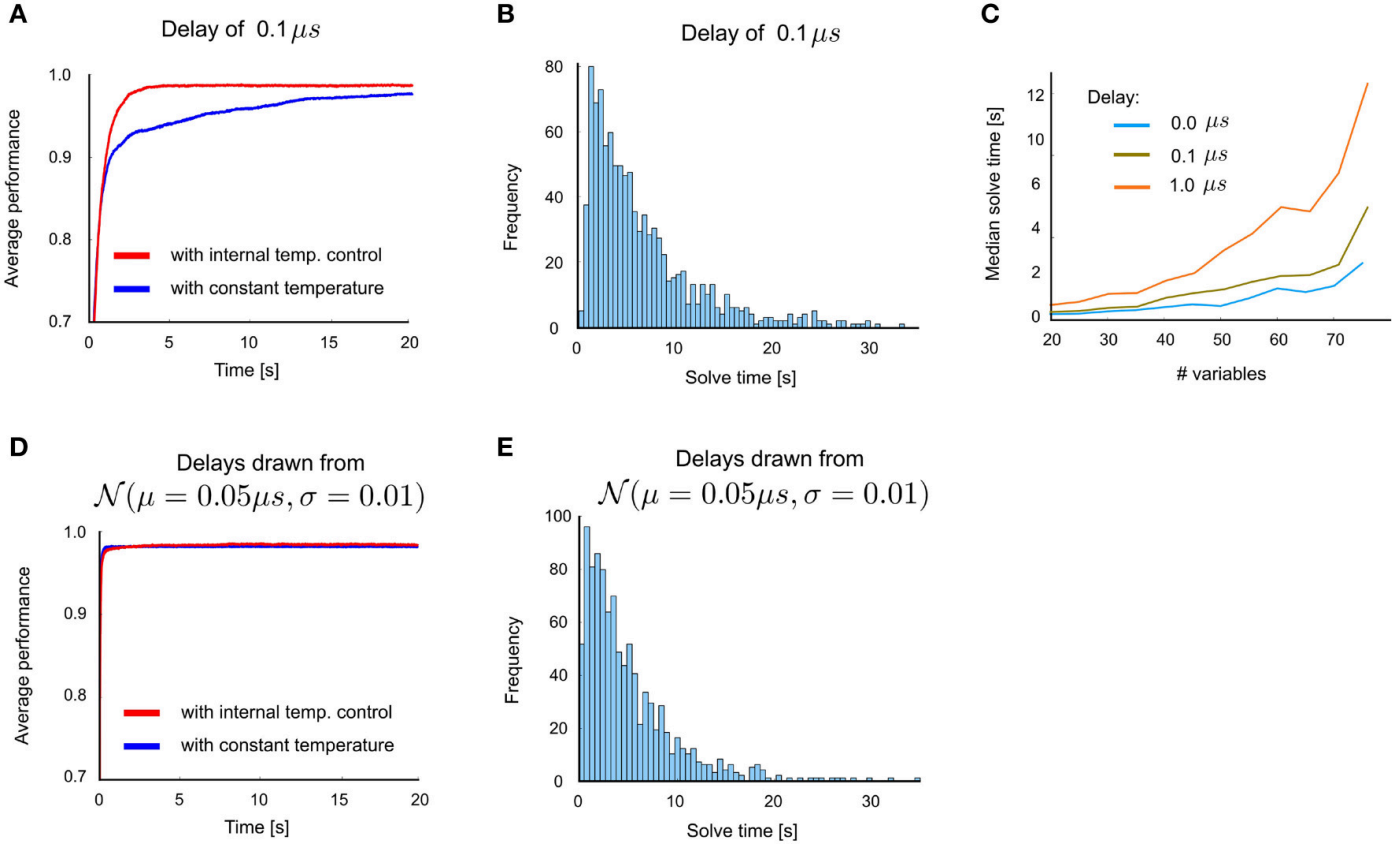
**Influence of transmission delays between neurons on network performance for 3-SAT**. **(A)** Performance with and without internal temperature control (average over 100 runs), with uniform delay 0.1μ*s*. **(B)** Distribution of solve times with uniform delay 0.1μ*s*. **(C)** Comparison of median solve times for different problem sizes, using delays 0∕0.1∕1μ*s*. **(D)** Network performance with non-uniform delays, where the transmission delay of each connection is drawn from a Gaussian distribution N(μ = 0.05μ*s*, σ = 0.01). This plot suggests that primarily the maximum delay time matters, whereas non-uniform delay times cause no additional problem. **(E)** Distribution of solve times with these randomly drawn delays is similar to that with uniform delays in panel **B**.

The simulation results described above were obtained in the absence of transmission delays. Figures 5A–C summarize the impact on performance when uniform delays are introduced into the network architecture of Figure 4A. For a small delay of 0.1 μs, only a mild performance degradation is observed: compare Figures 5A–C with Figures 4D–F. Computations times remain in the same order of magnitude as with the zero-delay case for delays up to 1 μs (Figure 5C). Larger delays were observed to lead to substantial performance reduction. This arises from differences in firing times of different neurons that are smaller than the chosen delay.

In order to test whether uniformity of delays is essential for the computational performance we also investigated the impact of non-uniform delays, where delays were randomly and independently drawn from a normal distribution with μ = 0.05μ*s* and σ = 0.01 truncated at 0 and 0.1 μs (Figures 5D,E). Our results suggest that a variability of delays does not give rise to an additional degradation of performance, only the maximal delay appears to matter. Altogether we have shown that transmission delays of more than 0.1 μs impair the computational performance. But spike transmission within 0.1 μs can easily be achieved in electronic hardware.

Spike transmission in biological networks of neurons takes at least a few ms. Hence if brains make use of stochastic sampling, they would have to slow down the sampling speed. They could sample for example from network states that are defined by the set or sequence of neurons that fire during one cycle of some background oscillation (as in Jezek et al., 2011). In this way longer transmission delays can be tolerated because it approximately suffices if the spike of a neuron reaches other neurons to which it is synaptically connected within the same cycle. Similar ideas have recently been explored for neuromorphic hardware (Mostafa et al., 2015b)

## 3. Discussion

We have presented a theoretical basis and four rules, illustrated in Figure 1, for designing networks of spiking neurons which can solve complex constraint satisfaction problems in an efficient manner. One new feature of our approach is to design networks of spiking neurons not on the basis of desirable signal cascades and computational operations on the level of neurons and neural codes. Rather, we propose to focus immediately on the network level, where the probability distribution of spike-based states of the whole network (or in alternative terminology: the energy landscape of spike-based network states) defines an alternative conceptual and mathematical level on which the network can be programmed, or taught, if one considers a learning approach. We have demonstrated that properties of the energy function of the network can easily be programmed through modular design, making use of network motifs that each contribute particular identifiable features to the global energy function. A principled understanding of the interaction between local network motifs and global properties of the energy function of the network is provided by a Modularity Principle (Theorem 1). The local network motifs that we consider can be seen as analogs to the classical computing primitives of deterministic digital circuits (Boolean gates) for stochastic spike-based computations.

The resulting spike-based networks differ from previously considered ones in that they use noise as computational resource. Without noise, there is no stationary distribution of network states and no energy function. A surprising feature of the resulting stochastic networks is that they benefit in spite of the noise from the possibility to use the timing of spikes as a vehicle for encoding information during a computation. The temporal order of spikes does not directly become computationally relevant, rather coincidences and almost-coincidences of spikes from specific sets of neurons within a short time window. This holds in spite of the fact that they operate in continuous time. It arises from the fact that we define network states (see Figure 1A) by recording which neuron fires within a short time window.

Finally, we have addressed the question whether there are cases where spike-based computation is faster than a corresponding non-spiking computation. We have shown in Figure 3 that this is the case for the two TSP instances that we have considered. The TSP is a particularly suitable task for such comparison, since it can be solved also by a Boltzmann machine with exactly the same architecture. In fact, we have considered for comparison a Boltzmann machine that has in addition the same energy function (or equivalently: the same stationary distribution of network states) as the spiking network. We have shown that the sampling dynamics of the spiking network is fundamentally different, since it has an inherent mechanism for bypassing high energy barriers in the search for a state with low energy.

The Traveling Salesman Problem is among the most well-known combinatorial optimization problems (Cook et al., 1998) and has been studied intensely for both theoretical and practical reasons: TSP belongs to the class of NP-hard problems, and hence no polynomial-time algorithm is known for solving TSP instances in general. Nevertheless, TSPs arise in many applications, e.g., in logistics, genome sequencing, or the efficient planning of laser positioning in drilling problems (Applegate et al., 2011). Substantial efforts have been invested in the development of efficient approximation algorithms and heuristics for solving TSP instances in practice. In the Euclidean (planar) case, for example, where movement costs *c*_*ij*_ correspond to Euclidean distances between cities in a two-dimensional plane, a polynomial-time approximation scheme (PTAS) exists which is guaranteed to produce approximate solutions within a factor of (1 + ϵ) of the optimal tour in polynomial time (Arora, 1998). Various other heuristic algorithms for producing approximate or exact solutions (with typically weaker theoretical support) are often successfully used in practice (Applegate et al., 2011). An implementation for solving TSPs with artificial non-spiking neurons was first provided in the seminal paper by Hopfield and Tank (1986). Malaka and Buck (2000) ported their approach to deterministic networks of spiking neurons and reported that such networks found an optimal solution for a planar TSP instance with 8 cities. We are not aware of previous work on solving TSP instances with stochastic spiking neurons.

Substantial efforts have been invested in the development of efficient approximation algorithms and heuristics for solving TSP instances in practice. In the Euclidean (planar) case, for example, a polynomial-time approximation scheme (PTAS) exists which is guaranteed to produce approximate solutions within a factor of (1 + ϵ) of the optimal tour in polynomial time (Arora, 1998). Various other heuristic algorithms with weak theoretical support for producing approximate or exact solutions are often successfully used in practice. One of the most efficient heuristic solvers producing exact solutions for practical TSP problems is CONCORDE (Applegate et al., 2011). An implementation for solving TSPs with artificial neurons was first provided in the seminal paper of Hopfield and Tank (1986). The neurons in this model were deterministic and analog. Due to deterministic dynamics it was observed that the network often got stuck in infeasible or suboptimal solutions. Various incremental improvements have been suggested to remedy the observed shortcomings (Van den Bout and Miller III, 1989; Chen and Aihara, 1995).

We have shown that the same design principles can also be applied to solve 3-SAT with spiking neurons. 3-SAT is the problem of determining if a given Boolean formula in conjunctive normal form (i.e., conjunctions of disjunctive clauses) with clauses of length 3 is satisfiable. 3-SAT is NP-complete, i.e., there are no known methods that can efficiently solve general 3-SAT problems. The NP-completeness of 3-SAT (and general SATISFIABILITY) was used by Karp to prove NP-completeness of many other combinatorial and graph theoretical problems (Karp's 21 problems, Karp, 1972). Large satisfiability problems appear in many practical applications such as automatic theorem proving, planning, scheduling and automated circuit design (Biere, 2009).

Despite exponential worst-case complexity, many problems arising in practice can be solved quite efficiently by clever (heuristic) algorithms. A variety of algorithms have been proposed (Gomes et al., 2008). Modern SAT solvers are generally classified in complete and incomplete methods. Complete solvers are able to both find a satisfiable assignment if one exists or prove unsatisfiability otherwise. Incomplete solvers rely on stochastic local search and thus only terminate with an answer when they have identified a satisfiable assignment but cannot prove unsatisfiability in finite time. The website of the annual SAT-competition, http://www.satcompetition.org/, provides an up-to-date platform for quantitative comparison of current algorithms on different subclasses of satisfiability problems.

A very interesting method for solving SATISFIABILITY on neuromorphic chips, but with oscillators instead of spiking neurons, has very recently been proposed by Mostafa et al. (2015a). They replace standard pseudo random number generators by using non-repeating phase relations among incommensurable analog oscillators to force the network to continuously explore the solution space. In order to motivate their alternative to statistical mechanics approaches, they point out that “while noise is an inextricable part of any physical system, controlling its power to balance ‘exploratory’ vs. ‘greedy’ search …puts an additional overhead on the physical implementation.” It is clear that noise control would be needed for simulated annealing approaches. But the methods discussed in this article do no require an external control of the power of noise. In addition our approach can be based on a rigorous mathematical theory.

We have not addressed the question of learning. There is a rich literature on learning for Boltzmann machines and for networks of stochastic spiking neurons. In particular, a model for learning arbitrary discrete probability distributions from examples is proposed in forthcoming work (Pecevski and Maass, in press). But we are not aware of compelling ideas for the learning of constraints in either type of network. One could engage supervised learning or use reinforcement learning methods for learning salient constraints from trial and error. But this is not likely to be the only way how constraints get encoded by networks of neurons in the brain. Some work in cognitive science suggests that some fundamental constraints that are relevant for motor planning, perception, and other constraint satisfaction tasks that a human brain has to solve are innate, or were previously learned for a related task and transferred to a new task setting (Tenenbaum et al., 2011). Hence the challenge to provide a model for learning of constraint satisfaction problems in neural networks appears to be of high interest, but not straightforward.

Altogether we have shown that there is a rich world of design methods and algorithmic approaches for networks of spiking neurons with noise that remains to be explored. We have discussed in this article only applications to two NP-hard constraint satisfaction problems that represent well known benchmark tasks. But obviously there are many other constraint satisfaction problems that arise in the interpretation of ambiguous signals, multi-sensory processing, planning, optimization, and problem solving. Some of these problems are also NP-hard, but there are also many practically important constraint satisfaction problems that are not NP-hard. We conjecture that a large number of these problems can be solved efficiently, in particular with low energy consumption if implemented in neuromorphic hardware, by networks of spiking neurons.

## 4. Methods

### 4.1. Details to the TSP application (Figure 2)

For a general TSP problem described by a set of *N* nodes and *N* · (*N* − 1) edges with associated costs *c*_*ij*_ to move from one node to another, the goal is to find a tour with minimum cost which visits each node exactly once. When the cost *c*_*ij*_ to move from node *i* to node *j* equals the cost *c*_*ji*_ from *j* to *i* for all pairs of nodes then the problem is called symmetric. If movement costs *c*_*ij*_ are Euclidean distances between cities in a two-dimensional plane, the TSP problem is called Euclidean or planar.

#### 4.1.1. Network architecture for solving TSP

In Figure 2 we demonstrate an application of our theoretical framework for stochastic spike-based computation to the TSP. To encode the node (city) visited at a given step of a tour in a TSP problem consisting of *N* nodes, a discrete problem variable with *N* possible values is needed. To encode a tour with *N* steps, *N* such problem variables are required. More precisely, for efficiency reasons we consider in our approach a relaxed definition of a tour: a tour for an *N*-node problem consists of *N*′ = *N* + *N*_resting_ steps (and problem variables). A tour is valid if each node is visited at least once. Since there are more steps than nodes, some nodes have to be visited twice. We require that this can only occur in consecutive steps, i.e., a the salesman may remain for at most one step in a city before he must move on. We observed that using such relaxed definition of a tour with *N*_resting_ additional “resting” steps may considerably improve the efficiency of the stochastic search process.

To solve a TSP problem one then needs to consider three types of constraints:

- Each of the *N*′ problem variables should have at any moment a uniquely defined value assigned to it.
- Each value from the problem variable domain {1, …, *N*} must appear at least once in the set of all problem variables (each node has to be visited at least once). At the same time only neighboring problem variables, i.e., those coding for consecutive steps, may have the identical values (allowing for “resting” steps).
- The penalty that two consecutive problem variables appear in a given configuration, i.e., a particular transition from node *i* to some other node *j*, must reflect the traveling cost between the pair of nodes.

In the spiking network implementation, each step (problem variable) *n* ∈{1, … *N*′} is represented by a set of *N* principal neurons, ν_*n*1_, …, ν_*nN*_, i.e., each principal neuron represents one of the *N* nodes (cities). A proper representation of problem variables (and implementation of constraints (a)) is ensured by forming a WTA circuit from these *N* principal neurons which ensures that most of the time only one of these principal neurons is active, as described in Supplementary Material Section 3.2: The WTA circuit shapes the energy function of the network such that it decreases the energy (increases probability) of states where exactly one of the principal neurons in the WTA is active, relative to all other states. If the WTA circuits are setup appropriately, most of the time exactly one principal neuron in each WTA will be active. The identity of this active neuron then naturally codes for the value of the associated problem variable. Hence, one can simply read out the current assignment of values to the problem variables from the network principal state based on the activity of principal neurons within the last τ time units.

The parameters of the WTA circuits must be chosen appropriately to ensure that problem variables have a properly defined value most of the time. To achieve this, the inhibitory auxiliary neuron is equipped with a very low bias *b*_*inh*_, such that the inhibition and therefore the WTA mechanism is triggered only when one of the principal neurons spikes. The inhibitory neuron is connected to all principal neurons associated with the considered problem variable through bidirectional connections *w*_*WTA*_ and *w*_*exc*_ (from and to the inhibitory neuron, respectively). The inhibition strength *w*_*WTA*_ should be chosen strong enough to prevent all principal neurons in the WTA circuit from spiking (to overcome their biases) and *w*_*exc*_ should be set strong enough so that it activates inhibition almost immediately before other principal neurons can spike. At the same time the biases of the principal neurons *b*_*WTA*_ should be high enough to ensure sufficient activation of principal neurons within the WTA circuit so that the corresponding problem variable is defined most of the time. To enforce that a specific problem variable assumes a particular value one can modulate the biases of the associated principal neurons and thereby decrease the energy of the desired problem variable value. We used this to set the value of the first problem variable *n* = 1 to point to the first node (city). In particular, the bias of the principal neuron ν_11_ is set to *b*_*P*_ (should be set high enough to ensure exclusive activity of that neuron) and biases of all other principal neurons in the first WTA circuit are set to *b*_*N*_ (should be set low enough to prevent neurons from being active).

The implementation of constraints (b) requires that all problem variables have different values except if they are neighboring variables. This is realized by connecting bidirectionally all principal neurons which code for the same value/node in different WTA circuits with strong negative connection *w*_*unique*_, except if WTA circuits (or corresponding problem variables) are neighboring, i.e., represent adjacent steps of a tour. This simply increases the energy and decreases the probability of network states where the principal neurons coding for the same city in different WTAs are co-active, except if they are in neighboring WTAs.

Finally, constraints (c) are implemented by adding synaptic connections between all pairs of principal neurons located in neighboring problem variables, except between principal neurons coding for the same value. A positive connection between two principal neurons increases the probability that these two neurons become co-active (the energy of corresponding network states is decreased), and vice versa. Hence, when movement costs between two nodes (cities) *i* and *j* at steps *n* and *n* + 1, respectively, are low, the corresponding neurons should be linked with a positive synaptic connection in order to increase the probability of them becoming active together. We chose to encode weights such that they reflect the relative distances between cities. To calculate the weights we normalize all costs of the TSP problem with respect to the maximum cost, yielding normalized costs ${\tilde{c}}_{ij}\in [0,1]$. Then we rescale and set the synaptic connections between neuron ν_*ni*_ and ν_(*n* + 1)*j*_ according to

(15) $$w_{ni,(n+1)j}=w_{(n+1)j,ni}=w_{offset}+(1-{\tilde{c}}_{ij})*w_{scale}$$

for *n* ∈{1, … *N*′ − 1}. The connections between step *N*′ and step 1 are set in an analogous fashion. As a result of this encoding, the energy landscape of the resulting network assigns particularly low energies to network states representing low-cost tours.

The proposed neural network architecture for solving TSP results in a ring structure (as the last and the first problem variable are also connected). The architecture requires (*N* + 1)(*N* +*N*_*resting*_) neurons (including inhibitory neurons in WTA circuits) and *N*(*N* +*N*_resting_)(2*N* + *N*_resting_ − 2) number of synapses.

#### 4.1.2. Further parameters and details for the TSP application

The planar TSP in 2Ais taken from http://www.math.uwaterloo.ca/tsp/world/countries.html. The 38 nodes in the problem correspond to cities in Djibouti, and movement costs are Euclidean distances between cities. To solve the problem we used the following setup: PSP length τ = 10*e* − 3 and refractory period of 10ms for all neurons, *b*_*WTA*_ = −0.45, *b*_*P*_ = 100, *b*_*N*_ = −100, *b*_*inh*_ = −10, *w*_*WTA*_ = −100, *w*_*exc*_ = 100, *w*_*unique*_ = −14.7, *w*_*scale*_ = 19.4, *w*_*offset*_ = −5 and *N*_resting_ = 7, resulting in the neural network consisting of 1755 neurons. The value of the first variable (the first step) was fixed to the first city. For the asymmetric TSP problem, in order to facilitate comparison we chose a similarly sized problem consisting of 39 cities from TSPLIB, http://comopt.ifi.uni-heidelberg.de/software/TSPLIB95/, file ftv38.atsp.gz. To solve this asymmetric TSP problem we used the same architecture as above, with slightly different parameters: *b*_*WTA*_ = 1.3, *w*_*unique*_ = −14.1, *w*_*offset*_ = −7.9, *w*_*scale*_ = 20.8, *N*_resting_ = 8, resulting in a neural network consisting of 1880 neurons.

Reading out the current value assignment of each problem variable is done based on the activity of the principal neurons which take part in the associated WTA circuit. The performance of the network is calculated at any moment as the ratio of the optimal tour length and the current tour length represented by the network. In order for the currently represented tour to be valid all variables have to be properly defined and each value (city) has to appear at least once. Although this is not *always* the case, exceptions occur rarely and last for very short periods (in the order of ms) and therefore are not visible in the performance plots.

The significance of the empirically found differences in the performance of spiking networks and Boltzmann machines for the two TSP instances, shown in Figures 3E,F, was evaluated with a two-sample KolmogorovSmirnov test, which checks if two sample sets are drawn from the same distribution. All *p*-values were < 1%. The precise values were for the symmetric problem at cost = 10,000: p-value = 1.4 × 10^−31^ (#samples BM = 98 NS = 100), for the symmetric problem at cost = 8500: p-value = 7.5 × 10^−9^ (#samples BM = 63 NS = 100), for the asymmetric problem at cost = 2200: *p*-value = 3.0 × 10^−16^ (#samples BM = 100 NS = 100), for the asymmetric problem at cost = 1800: p-value = 3.5 × 10^−10^ (#samples BM = 77 NS = 100). Differences in the number of samples (#samples) arose because the Boltzmann machines did not find the solution to the problem in every run during the simulation time (100,000 state changes).

### 4.2. Details to the 3-SAT application (Figure 4)

#### 4.2.1. Network architecture for solving 3-SAT

For the demonstration in Figure 4 we consider satisfiable instances of random 3-SAT problems with a clause to variable ratio of 4.3 near the crossover point. Based on our theoretical framework a network of stochastic spiking neurons can be constructed which automatically generates valid assignments to 3-SAT problems by stochastic search (thereby implementing an incomplete SAT solver). The construction based on WTA and OR circuits is quite straightforward: each Boolean problem variable of the problem is represented by a WTA circuit with two principal neurons (one for each truth value). Analogous to the TSP application, the WTA circuits ensure that most of the time only one of the two principal neurons in each WTA is active. As a result, most of the time a valid assignment of the associated problem variables can be read out.

Once problem variables are properly represented, clauses can be implemented by additional OR circuits. First, recall that, in order to solve a 3-SAT problem, all clauses need to be satisfied. A clause is satisfied if at least one of its three literals is true. In terms of network activity this means that at least one of the three principal neurons, which code for the involved literals in a clause, needs to be active. In order to encourage network states in which this condition is met, one may use an OR circuit motif which specifically increases the energy of network states where none of the three involved principal neurons are active (corresponding to assignments where all three literals are false). Such application of an OR circuit will result in an increased probability for the clause to be satisfied. If one adds such an OR circuit for each clause of the problem one obtains an energy landscape in which the energy of a network state (with valid assignments to all problem variables) is proportional to the number of violated clauses. Hence, stacking OR circuits implicitly implements the conjunction of clauses.

The described network stochastically explores possible solutions to the problem. However, in contrast to a conventional computer algorithm with a stopping criterion, the network will generally continue the stochastic search even after it has found a valid solution to the problem (i.e., it will jump out of the solution again). To prevent this, based on feedback signals from WTA and OR circuits it is straightforward to implement an internal temperature control which puts the network in a low temperature regime once a solution has been found (based on feedback signals). This mechanism basically locks the network into the current state (or a small neighboring region of the current state), making it very unlikely to escape. The mechanism is based on a internal temperature control neuron which receives feedback signals from WTA and OR circuits (signaling that all the constraints of the problem were met) and which activates additional circuits (copies of OR circuits) that effectively put the network in the low temperature regime.

#### 4.2.2. Parameters and further details for the 3-SAT application

Each OR circuit consists of two auxiliary neurons, I and II, with biases of 0.5*B* and − 3.5*B*, respectively, where *B* is some constant. Both auxiliary neurons connect to those principal neurons which code for a proper value of the literals in the clause (in total to 3 neurons), using synaptic connections with weights *w*_*OR*_ and −*B* (from and to the auxiliary neuron I, respectively), and −w_*OR*_ and *B* (from and to the auxiliary neuron II, respectively). Additionally, the auxiliary neuron I connects to the auxiliary neuron II with the strength 3*B*. Here the strength of incoming connections from principal neurons and the bias of auxiliary neuron I are set such that its activity is suppressed whenever at least one of three principal neurons is active, while parameters for auxiliary neuron II are set such that it can be activated only when auxiliary neuron I and at least one of three principal neurons are active together (see OR circuit motif for more details).

The internal temperature control mechanism is implemented as follows. The regime of low temperature is constructed by duplicating all OR circuits—so by adding for each clause two additional auxiliary neurons III and IV which are connected in the same way as the auxiliary neurons I and II (they target the same neurons and are also connected between themselves in the same manner) but with different weights: w_*OR*2_ and −*B* (from and to auxiliary neuron), and −w_*OR*2_ and *B* (from and to auxiliary neuron), for the auxiliary neurons III and IV, respectively. The use of the same OR motif ensures the same functionality, which is activated when needed in order to enter the regime of low temperature and deactivated to enter again regime of high temperature. The difference in connections strength between *w*_*OR*1_ and *w*_*OR*2_ determines the temperature difference between high and low temperature regimes. In addition the biases of additional auxiliary neurons are set to − 0.5*B* and −6.5*B* (III and IV aux. neuron), so that they are activated (i.e., functional) only when a certain state (the solution) was detected. This is signaled by the global temperature control neuron with bias *b*_*glob*_ that is connected to both additional auxiliary neurons of all clauses with connection strengths *B* and 3B to the III and IV auxiliary neuron, respectively, so that once the global temperature control neuron is active the additional auxiliary neurons behave exactly as auxiliary neurons I and II. Additionally, the global temperature control neuron is connected to every other principal neuron with connection strength *w*_*glob*_, which mimics the change in temperature regime in WTA circuits. The global neuron is active by default, which is ensured by setting the bias *b*_*glob*_ sufficiently high, but is deactivated whenever one of the status neurons, which check if a certain clause is not satisfied, is active (*not OK* signals). There is one status neuron for each clause, with bias set to −2.5*B*, where each one of them receives excitatory connections of strength *B* from all neurons not associated with that particular clause. Therefore, if all problem variables that participate in the clause are set to the wrong values, this triggers the status neuron which reports that the clause is not satisfied. This automatically silences the global neuron signaling that the current network state is not a valid solution. Note that implementation of such internal temperature control mechanism results with inherently non-symmetric weights in the network.

For described architecture of a spiking neural network the total number of neurons needed is 3*N* + 2*M* (2 + 1 per WTA circuit, and 2 per OR circuit), while the number of connections is 4*N* + 13*M*. Notably, both the number of neurons and the number of synapses depend linearly on the number of variables *N* (the number of clauses linearly depends on the number of variables if problems with some fixed clauses-to-variables ratio are considered). To implement in addition described internal temperature control mechanism one needs additional 3*M* + 1 neurons and 2*N* + 20*M* synapses.

The architecture described above was used in simulations with the following parameters: τ = 10*e* −3 and refractory period of 10ms for all neurons except for the global neuron which has τ = 9*e* −3 and refractory period of 9ms, b_*WTA*_ = 2, *b*_*inh*_ = −10, *b*_*glob*_ = 10, *B* = 40, *w*_*WTA*_ = −100, *w*_*exc*_ = 100, *w*_*OR*1_ = 2.5, *w*_*OR*2_ = 10, with rectangular PSPs of 10ms duration without transmission delays for all synapses except for the one from the global neuron to the additional auxiliary neurons where the PSP duration is 11ms. The network which solves the considered Boolean problem in Figure 4A consisting of 50 variables and 218 clauses has 586 neurons.

To calculate the performance of a solution at any point in time we use as a performance measure the ratio between the number of satisfied clauses and total number of clauses. If none of the variables which take part in a clause are properly defined then that clause is considered unsatisfied. As a result, this performance measure is well-defined at any point in time.

For the analysis in Figure 4F of problem size dependence we created random 3-SAT problems of different sizes with clause-to-variable ratio of 4.3. To ensure that a solution exists, each of the created problems was checked for satisfiability with zhaff, a freely available (complete) 3-SAT solver.

Finally, note that the proposed architecture can be used in analogs way in order to solve k-SAT problems, where each clause is composed of *k* literals. In this case the only change concerns OR circuit motifs which now involve for each clause *k* associated principal neurons.

### 4.3. Software used for simulations

All simulations were performed in NEVESIM, an event-based neural simulator (Pecevski et al., 2014). The analysis of simulation results was performed in Python and Matlab.

## Author contributions

Conceived and designed the experiments: ZJ, SH, and WM. Performed the experiments: ZJ. Wrote the paper: ZJ, SH, and WM. Theoretical analysis: SH and WM.

## Funding

The work was partially supported by the European Union project #604102 (Human Brain Project).

### Conflict of interest statement

The authors declare that the research was conducted in the absence of any commercial or financial relationships that could be construed as a potential conflict of interest.
