# Supplementary material for: Solving Constraint Satisfaction Problems with Networks of Spiking Neurons
Source: Front Neurosci. 2016 Mar 30;10:118. doi: 10.3389/fnins.2016.00118 (PMC4811945; doi:10.3389/fnins.2016.00118)
Supplement: Supplementary file 1 [file Presentation1.PDF]

frontiers

Supplementary Material: Solving difficult computational tasks with spiking neurons

Zeno Jonke [1,†], Stefan Habenschuss [1,†] and Wolfgang Maass [1,*]

[†] *These authors have contributed equally to this work.*

Correspondence*:
Wolfgang Maass
Institute for Theoretical Computer Science, Graz University of Technology,
Inffeldgasse 16b/1, Graz, 8010, Austria, maass@igi.tugraz.at

1 STOCHASTIC NEURON MODEL

Neurons are modeled as simple stochastic point neurons with absolute refractory period τ. When not in a refractory state, neuron k spikes at an instantaneous firing rate which depends exponentially on the membrane potential $u_k(t)$ given by equation (M2)[1], according to,

$$\lim_{\delta t \to 0} p(\text{ neuron } k \text{ fires in } (t, t+\delta t]\,)/\delta t = \rho_k(t) = \frac{1}{\tau}\exp(u_k(t)) \quad , \tag{1}$$

with $\tau = 10$ms unless otherwise stated. An exponential dependence of a neuron's firing probability on the membrane potential has been suggested by **Jolivet et al.** (2006) based on a fit to experimental data. Similar stochastic neuron models have been suggested by **Truccolo et al.** (2005); **Buesing et al.** (2011).

Note that an exponential firing function is required for correct sampling when a constant "off-rate" is assumed (corresponding to a constant PSP length which does not depend on the pre-synaptic membrane potential). In the discrete-time variant of neural sampling **Buesing et al.** (2011), the resolution (how many discrete time steps constitute a refractory period) determines whether the canonical activation function resembles more a sigmoid or an exponential function. In discrete time implementations of neural sampling it may be thus expected that a variety of intermediate behaviors between Gibbs sampling and continuous-time neural sampling is found at different resolutions.

2 DETAILS TO PRINCIPLE 1: STATIONARY DISTRIBUTIONS AND ENERGY FUNCTIONS

2.1 NETWORK STATES

The state $x_k(t)$ of a principal neuron k at time t is defined as,

$$x_k(t) = \begin{cases} 1, & \text{if neuron k fired within } (t-\tau, t] \;\; , \\ 0, & \text{otherwise} \;\; , \end{cases} \tag{2}$$

where τ is a brief time window corresponding to the duration of a PSP. The state vector of all principal neurons (the *principal network state*) is denoted by $\mathbf{x}(t) = (x_1(t), \ldots, x_N(t))$. Unless otherwise stated, the term *network state* refers to the principal network state.

[1] We use prefix M to refer to the main text material.

The state $\xi_m(t)$ of an auxiliary neuron m is defined in an analogous manner to equation (2). The state vector of all auxiliary neurons is written as $\boldsymbol{\xi}(t) = (\xi_1(t), \ldots, \xi_M(t))$. The *full network state* is given by,

$$(\mathbf{x}(t), \boldsymbol{\xi}(t)) = (x_1(t), \ldots, x_N(t), \xi_1(t), \ldots, \xi_M(t)) \tag{3}$$

Similar notions of network state have been suggested by a number of experimental and theoretical papers **Schneidman et al.** (2006); **Berkes et al.** (2011); **Buesing et al.** (2011); **Pecevski et al.** (2011) and **Habenschuss et al.** (2013).

2.2 CONVERGENCE TO STATIONARY DISTRIBUTION

Under mild conditions, activity in a general spiking network with noise can be theoretically guaranteed to converge exponentially fast to a unique stationary distribution $p(\mathbf{x}, \boldsymbol{\xi})$ of full network states **Habenschuss et al.** (2013), regardless of initial network conditions. In the context of the stochastic neuron model , equations (M1)-(M2), it can be easily verified that the theoretical conditions for convergence are fulfilled if all weights are finite. Clearly, throughout the paper this condition is met. Exponentially fast convergence to a unique *marginal* distribution $p(\mathbf{x})$ over principal network states is a simple corollary that follows from the convergence to a unique joint distribution $p(\mathbf{x}, \boldsymbol{\xi})$.

2.3 ENERGY FUNCTIONS

In analogy with statistical physics **Plischke and Bergersen** (2006), we define the energy function $E(\mathbf{x})$ of a network of spiking neurons with a unique stationary distribution $p(\mathbf{x})$ of principal network states $\mathbf{x}$ as

$$E(\mathbf{x}) = -\log p(\mathbf{x}) + C \quad , \tag{4}$$

with an arbitrary constant C. The stationary distribution $p(\mathbf{x})$ can then be expressed as,

$$p(\mathbf{x}) = \frac{e^{-E(\mathbf{x})}}{\sum_{\mathbf{x}'} e^{-E(\mathbf{x}')}} \quad . \tag{5}$$

Note that according to this definition, energies are defined only up to a constant (a global shift applied to all states). To indicate that two energy functions are identical except for a constant shift we use the notation $E_1(\mathbf{x}) \triangleq E_2(\mathbf{x})$, i.e.

$$E_1(\mathbf{x}) \triangleq E_2(\mathbf{x}) \quad \Leftrightarrow \quad \exists C{\in}\mathbb{R}\ \forall \mathbf{x}\ \ (E_1(\mathbf{x}) = E_2(\mathbf{x}) + C) \quad . \tag{6}$$

3 DETAILS TO PRINCIPLE 2: SHAPING THE ENERGY FUNCTION THROUGH CIRCUIT MOTIFS

A key theoretical question is how the energy function $E(\mathbf{x})$ (or equivalently $p(\mathbf{x})$) over principal network states $\mathbf{x}$ depends on the parameters of a network, in particular on synaptic weights w_{kl} and neuronal excitabilities b_k among principal neurons, as well as on auxiliary circuits connected to the principal neurons. Previous work had shown that pair-wise symmetric connections between neurons map onto second-order dependencies between variables **Buesing et al.** (2011). **Pecevski et al.** (2011) demonstrated in addition how more complex dependencies can be encoded through the use of pre-processing circuits in the context of probabilistic inference.

Here we consider how in addition to second-order dependencies, common higher-order constraints of hard computational problems can be encoded through the use of simple auxiliary circuit motifs, in a manner suitable for modularity and large-scale circuit design.

3.1 CONDITIONS FOR MODULARITY

To facilitate systematic design of complex energy landscapes, we would like to find a basic set of auxiliary circuit motifs which can be combined in arbitrarily rich ways with predictable outcomes. A particularly desirable feature to aim for is linear modularity, such that the energy contribution to the energy landscape of each circuit motif is independent of the presence of other circuit motifs.

The starting point for the following results is the Neural Computability Condition (NCC) from **Buesing et al.** (2011), which provides a sufficient condition for a network of model neurons given by equation (M1) to sample from some desired distribution $p(\mathbf{x})$. The NCC requires that the membrane potential of each neuron k obeys,

$$u_k(t) = \log \frac{p(x_k = 1|\mathbf{x}_{\setminus k}(t))}{p(x_k = 0|\mathbf{x}_{\setminus k}(t))} \quad . \tag{7}$$

where $\mathbf{x}_{\setminus k}(t)$ denoting the current state vector of all principal neurons excluding neuron k. In terms of a desired energy function $E(\mathbf{x})$, the NCC can be reformulated as

$$u_k(t) = E(x_k = 0, \mathbf{x}_{\setminus k}(t)) - E(x_k = 1, \mathbf{x}_{\setminus k}(t)) \quad , \tag{8}$$

where we use the simplified notation $E\left(x_k = \cdot, \mathbf{x}_{\setminus k}(t)\right) := E(x_1(t), \ldots, x_{k-1}(t), \cdot, x_{k+1}(t), \ldots, x_N(t))$.

In the absence of auxiliary neurons/circuits it was shown by **Buesing et al.** (2011) that, if all synaptic connections among principal neurons are symmetric ($w_{kl} = w_{lk}$), a network $\mathcal{N}$ of principal neurons will sample from a Boltzmann distribution with energy function given by equation (M3). Suppose that a set of auxiliary circuits $\mathcal{I}$ is added (and connected) to such a principal network. Then, due to linearity of membrane integration ,see equation (M2), the membrane potential of a principal neuron k in the presence of such auxiliary circuits can be written as equation (M7).

The energy contribution that each auxiliary circuit C_i makes to the energy function $E(\mathbf{x})$ of principal neurons, however, may be arbitrarily complex. In particular, it may depend in non-trivial ways on the presence and detailed structure of other auxiliary circuits and synaptic weights and biases of the principal network. Under appropriate conditions, however, the energy contribution of each auxiliary circuit becomes linear and independent of the remaining network (Theorem 1).

Theorem 1 suggests that auxiliary circuits should be constructed in a highly specific manner to support modularity. In particular, condition given by equation (M8) states that auxiliary circuit contributions to the membrane potential of a principal neuron k should be basically memoryless and reflect a specific function of the current state of the remaining network, $\mathbf{x}_{\setminus k}(t)$. Note that this function (the right-hand side of equation (M8)) has a very intuitive interpretation: a circuit C_i should inform each principal neuron k about the currently expected drop in the energy function U_i that can be achieved by a spike of neuron k (i.e. a switch from $x_k = 0$ to $x_k = 1$).

Proof of Theorem 1: If equation (M8) holds for all C_i then the membrane potential of a principal neuron k in the presence of some subset of auxiliary circuits $C_i, i \in \mathcal{I}$, is given at time t by,

$$u_{k,\mathcal{I}}(t) = b_k + \sum_{l=1}^{N} w_{kl}\, x_l(t) + \sum_{i \in \mathcal{I}} \left[U_i\left(x_k = 0, \mathbf{x}_{\setminus k}(t)\right) - U_i\left(x_k = 1, \mathbf{x}_{\setminus k}(t)\right) \right] \quad . \tag{9}$$

To verify that the network has stationary distribution

$$p(\mathbf{x}) \propto e^{-E_{\mathcal{N}}(\mathbf{x}) - \sum_{i \in \mathcal{I}} U_i(\mathbf{x})} \tag{10}$$

we plug into the NCC given by equation (7) from **Buesing et al.** (2011),

$$\log \frac{p(x_k = 1|\mathbf{x}_{\backslash k})}{p(x_k = 0|\mathbf{x}_{\backslash k})} = \log \frac{p(x_k = 1, \mathbf{x}_{\backslash k})}{p(x_k = 0, \mathbf{x}_{\backslash k})} \tag{11}$$

$$= \log p(x_k = 1, \mathbf{x}_{\backslash k}) - \log p(x_k = 0, \mathbf{x}_{\backslash k}) \tag{12}$$

$$= -E_{\mathcal{N},\mathcal{I}}(x_k = 1, \mathbf{x}_{\backslash k}) + E_{\mathcal{N},\mathcal{I}}(x_k = 0, \mathbf{x}_{\backslash k}) \tag{13}$$

$$= -E_{\mathcal{N}}(x_k = 1, \mathbf{x}_{\backslash k}) + E_{\mathcal{N}}(x_k = 0, \mathbf{x}_{\backslash k}) \tag{14}$$

$$+ \sum_{i \in \mathcal{I}} [-U_i(x_k = 1, \mathbf{x}_{\backslash k}) + U_i(x_k = 0, \mathbf{x}_{\backslash k})] \tag{15}$$

$$= b_k + \sum_{l=1}^{N} w_{kl} x_l + \sum_{i \in \mathcal{I}} [-U_i(x_k = 1, \mathbf{x}_{\backslash k}) + U_i(x_k = 0, \mathbf{x}_{\backslash k})] \ . \tag{16}$$

Thus, a network with membrane dynamics given by equation (9) meets the NCC for the distribution in equation (10). As a result, equation (10) must be the unique stationary distribution of the network, and the energy function is given by $E_{\mathcal{N}}(\mathbf{x}) + \sum_{i \in \mathcal{I}} U_i(\mathbf{x})$.
□

Note that, in contrast to neural sampling theory **Buesing et al.** (2011), Theorem 1 is concerned with the distribution over a subset of all neurons (the principal neurons $\mathbf{x}$), i.e. the marginal distribution $p(\mathbf{x})$ after integrating out all auxiliary variables $\boldsymbol{\xi}$. A concrete application of Theorem 1 is the design of auxiliary circuit motifs which *approximate* equation (M8) in practice, as described below for the WTA and OR motifs.

3.2 WTA CIRCUIT MOTIF

The WTA circuit motif consists of a single auxiliary neuron which is reciprocally connected to some subset $\mathcal{K} \subseteq \{1, \dots N\}$ of principal neurons (Fig. 1B, top left). The goal of the WTA motif is to achieve that most of the time *exactly* one neuron in $\mathcal{K}$ is active: whenever one principal neuron spikes it activates the (inhibitory) auxiliary neuron which suppresses all other principal neurons.

More precisely, in terms of energies, the goal of the WTA motif is to increase the energies of all network states with more or less than one active neuron in $\mathcal{K}$. This can be achieved in two steps. First, the energy of all network states where more than one neuron in $\mathcal{K}$ is active is increased. We found that this can be robustly achieved by a single inhibitory neuron which receives strong excitatory connections from $\mathcal{K}$, and sends strong inhibitory connections back to $\mathcal{K}$ (with some weight $w_{\text{WTA}} \ll 0$). The inhibitory neuron should have a low bias such that it only fires when one of the principal neurons is active. Second, the energy of states where no neuron in $\mathcal{K}$ is active is raised. This can be done most easily by raising the biases of all neurons in $\mathcal{K}$ by some constant b_{WTA} with $0 < b_{\text{WTA}} < -w_{\text{WTA}}$. Alternatively, this could in principle also be achieved by an additional auxiliary neuron which is constantly active and makes excitatory connections to all neurons in $\mathcal{K}$.

The design of the described implementation of the WTA circuit motif was based on the Modularity Principle (Theorem 1). This can be seen if one considers the desired energy function

$$U_{\text{WTA}[\mathcal{K}]}(\mathbf{x}) = \begin{cases} b_{\text{WTA}} \ , & \text{if } \sum_{k \in \mathcal{K}} x_k = 0 \ , \\ 0 \ , & \text{if } \sum_{k \in \mathcal{K}} x_k = 1 \ , \\ (-w_{\text{WTA}} - b_{\text{WTA}}) \cdot (-1 + \sum_{k \in \mathcal{K}} x_k) \ , & \text{if } \sum_{k \in \mathcal{K}} x_k > 1 \ . \end{cases} \tag{17}$$

Theorem 1 provides a concrete guideline for the design of an auxiliary circuit implementing this energy function. In particular, according to equation (M8) the ideal membrane potential contribution of the auxiliary circuit to principal neuron k, $\Delta u_{k,\text{WTA}[\mathcal{K}]}(t)$, for implementing equation (17) in a modular manner is given by,

$$\Delta u_{k,\text{WTA}[\mathcal{K}]}(t) = U_{\text{WTA}[\mathcal{K}]}(x_k = 0, \mathbf{x}_{\backslash k}) - U_{\text{WTA}[\mathcal{K}]}(x_k = 1, \mathbf{x}_{\backslash k}) \quad (18)$$

$$= \begin{cases} b_{\text{WTA}} \ , & \sum_{l \in \mathcal{K} \backslash k} x_l(t) = 0 \ , \\ b_{\text{WTA}} - w_{\text{WTA}} \ , & \sum_{l \in \mathcal{K} \backslash k} x_l(t) > 0 \ . \end{cases} \quad (19)$$

This membrane potential contribution is closely approximated by the described WTA circuit implementation: Regardless of the network state, there is a bias term b_{WTA} for each principal neuron $k \in \mathcal{K}$. As soon as one (or more) of these neurons fire, this triggers the auxiliary inhibitory neuron, which then strongly inhibits all competitors in $\mathcal{K}$ with weight w_{WTA}. The nature of the approximation lies mainly in the delay between the onset of activity of a winner and the onset of inhibition at the remaining principal neurons (due to stochastic firing of the auxiliary neuron).

3.3 OR CIRCUIT MOTIF

The OR circuit motif consists of two auxiliary neurons I and II reciprocally connected to some subset $\mathcal{K} \subseteq \{1, \ldots N\}$ of principal neurons (Fig. 1B, top right). The purpose of the OR motif is to ensure that most of the time *at least one* neuron in $\mathcal{K}$ is active. In essence, the auxiliary OR circuit motif remains silent as long as this OR-condition is met and at least one neuron in $\mathcal{K}$ is active. The motif is activated whenever it is detected that no neuron in $\mathcal{K}$ is active. The auxiliary circuit then excites the principal neurons until one of them fires.

At first sight, it may appear that this basic functionality should require only one auxiliary neuron, neuron I, which is inhibited by all principal neurons but starts firing immediately upon disinhibition when no principal neuron is active. Neuron I then keeps firing and exciting the principal neurons (with synaptic weight w_{OR}) until the OR-condition is restored.

However, a timing problem arises with this simple implementation of the OR motif. This is because once the OR-condition is restored, neuron I should be silenced immediately, along with all PSPs it is still causing in principal neurons. Clearly, the latter cannot be achieved by inhibiting neuron I because a spike of neuron I is an irreversible event, and PSPs, once elicited, have a fixed time course which cannot be stopped.

A refined implementation of the OR motif therefore contains in addition an auxiliary neuron II to mitigate this timing problem. Neuron II is activated precisely when PSPs of neuron I should be stopped: whenever some principal neuron just fired in response to neuron I, but the PSP of neuron I is still active in other neurons. Neuron II then immediately emits a spike which inhibits the principal neurons (with negative synaptic weight $-w_{OR}$), thereby canceling the effect of the prolonged PSPs of neuron I.

Analogous to the WTA circuit, the described implementation of the OR circuit motif aims to approximate the requirements of Theorem 1 for modularity. To see this, consider the energy function

$$U_{\text{OR}[\mathcal{K}]}(\mathbf{x}) = \begin{cases} 0 \ , & \text{if } \sum_{k \in \mathcal{K}} x_k \geq 1 \ , \\ w_{\text{OR}} \ , & \text{if } \sum_{k \in \mathcal{K}} x_k = 0 \ . \end{cases} \quad (20)$$

Using Theorem 1, according to condition given by equation (M8) this energy function can be implemented in a modular fashion by an auxiliary circuit making membrane potential contributions to each principal

neuron in $\mathcal{K}$,

$$\Delta u_{k,\mathrm{OR}[\mathcal{K}]}(t) = U_{\mathrm{OR}[\mathcal{K}]}(x_k = 0, \mathbf{x}_{\backslash k}) - U_{\mathrm{OR}[\mathcal{K}]}(x_k = 1, \mathbf{x}_{\backslash k}) \tag{21}$$

$$= \begin{cases} 0 \ , & \sum_{l \in \mathcal{K} \backslash k} x_l(t) \geq 1 \ , \\ w_{\mathrm{OR}} \ , & \sum_{l \in \mathcal{K} \backslash k} x_l(t) = 0 \ . \end{cases} \tag{22}$$

The OR circuit approximates this behavior as described above through the combination of two auxiliary neurons. The nature of the approximation is three-fold. First, when all principal neurons in an OR circuit have just turned off (and thus the constraint is not met anymore), the additional bias w_{OR} should ideally be communicated instantly to all neurons. However, the first auxiliary neuron fires in general with some small delay, and therefore the additional bias w_{OR} is signaled to the principal neurons slightly later than ideally required. Second, when a principal neuron eventually fires in response to the first auxiliary neuron, there is a delay until the second auxiliary neuron turns on to cancel the bias w_{OR} that is still present due to lingering PSPs from the first auxiliary neuron. Third, there is an "undershoot" effect when the excitatory PSP of the first auxiliary neuron has already vanished, but the inhibitory PSP of the second auxiliary neuron is still present. To minimize the error due to this effect, the overall biases of all principal neurons in an OR circuit should be kept high, in order to keep the typical delay between the activity onset of the first and the second auxiliary neuron as short as possible.

4 DETAILS TO PRINCIPLE 3: BENEFITS OF THE ASYMMETRY OF SPIKE-BASED STOCHASTIC SEARCH

Principles 1 and 2 pave the way towards massively parallel realizations of stochastic search in networks of spiking neurons. A first application of these principles has provided compelling results in simulations, as demonstrated in Fig. 2 and Fig. 4. A key theoretical question which then arises is to what extent different components of the system contribute to the observed performance. There are various aspects that can be examined in this context, such as the asynchronicity of message transfer, stochasticity, and the asymmetry of spike-based communication (a spike marks the onset of a fixed-length *on* period, whereas *off* periods vary randomly - hence *on* and *off* states are handled fundamentally different by a spiking network). We focus our analysis here on the role of the asymmetry of spike-based signaling, because its implications are arguably least well understood.

4.1 ASYMMETRIC VS. SYMMETRIC DYNAMICS

In order to isolate the effect of asymmetric signaling we consider an artificial non-spike-based "symmetrized" system in which *on* and *off* transitions of units are not mediated in an asymmetric fashion via spikes of fixed length, but rather in a symmetric manner. Specifically, we aim to morph neural spiking dynamics into the dynamics of Gibbs sampling **Bishop** (2006), one of the standard methods in statistics and machine learning for sampling from complex probability distributions. By theoretically analyzing and comparing the behavior of the two systems one can then reason about the specific role of asymmetric signaling.

A canonical way of symmetrizing the dynamics of a given spiking network with noise is to make sure that all other components and aspects of the system remain unchanged (event-based asynchronous signaling, stochasticity, synaptic weights and biases, definition of membrane potential u_k given the current *on*/*off* states of other neurons) and modify only the way the system handles transitions between *on* and *off* states. Importantly, to facilitate a comparison between asymmetric vs. symmetric dynamics, such modification should not alter the stationary distribution and energy function of the system.

For a stochastic spiking neuron embedded in some network, transitions occur from *off* to *on* states according to

$$\rho_{\text{on}}(u_k) = \frac{1}{\tau}\exp(u_k) \ , \tag{23}$$

whereas transitions from *on* to *off* occur deterministically after a period of τ time units has passed. Clearly, in a symmetric system transitions must occur stochastically in both directions (they cannot be both deterministic), with transition rates $\rho_{\text{on}}^{sym}(u_k)$ and $\rho_{\text{off}}^{sym}(u_k)$. Concrete symmetric expressions for $\rho_{\text{on}}^{sym}(u_k)$ and $\rho_{\text{off}}^{sym}(u_k)$ are obtained by using a continuous-time variant of Gibbs sampling **Bishop** (2006):

$$\begin{aligned}
\rho_{\text{on}}^{\text{sym}}(u_k) &= \rho_0 \cdot \frac{p(x_k = 1, \mathbf{x}_{\backslash k})}{p(x_k = 1, \mathbf{x}_{\backslash k}) + p(x_k = 0, \mathbf{x}_{\backslash k})} && (24)\\
&= \rho_0 \cdot \frac{1}{1 + p(x_k = 0, \mathbf{x}_{\backslash k})/p(x_k = 1, \mathbf{x}_{\backslash k})} && (25)\\
&= \rho_0 \cdot \left(1 + \exp\left(E(x_k = 1, \mathbf{x}_{\backslash k}) - E(x_k = 0, \mathbf{x}_{\backslash k})\right)\right)^{-1} && (26)\\
&= \rho_0 \cdot \sigma\left(E(x_k = 0, \mathbf{x}_{\backslash k}) - E(x_k = 1, \mathbf{x}_{\backslash k})\right) && (27)\\
&= \rho_0 \cdot \sigma(u_k) \ , && (28)\\
\rho_{\text{off}}^{\text{sym}}(u_k) &= \rho_0 \cdot \frac{p(x_k = 0, \mathbf{x}_{\backslash k})}{p(x_k = 1, \mathbf{x}_{\backslash k}) + p(x_k = 0, \mathbf{x}_{\backslash k})} && (29)\\
&= \rho_0 \cdot \left(1 + \exp\left(E(x_k = 0, \mathbf{x}_{\backslash k}) - E(x_k = 1, \mathbf{x}_{\backslash k})\right)\right)^{-1} && (30)\\
&= \rho_0 \cdot \sigma(-u_k) \ , && (31)
\end{aligned}$$

where $\sigma(u) = (1 + \exp(-u))^{-1}$ denotes the standard sigmoid function and ρ_0 is an arbitrary constant controlling the global speed of sampling. Such a continuous-time variant of Gibbs sampling has been proposed in the literature, for example, in the context of sampling from second-order Boltzmann machines **Yamanaka et al.** (1997).

4.2 ASYMMETRY FACILITATES TRANSITIONS ACROSS LARGE ENERGY BARRIERS

A somewhat unexpected difference which emerges from the comparative analysis between asymmetric and symmetric dynamics is that transitions across large energy barriers are much more likely and frequently to occur with asymmetric (spike-based) signaling. To see this, define the mean *on*-transition time $m_{\text{on}}(u)$ as the average time from the last *on*→ *off* transition until the next *off*→*on* transition, at a given membrane potential u. The mean *off*-transition time is defined in an analogous manner. In the stochastic spiking network these are given by,

$$\begin{aligned}
m_{\text{on}}(u) &= \frac{1}{\rho_{\text{on}}(u)} = \tau \cdot \exp(-u) \ , && (32)\\
m_{\text{off}}(u) &= \tau \ . && (33)
\end{aligned}$$

In the symmetric system, on the other hand, mean transition times in continuous time are given by,

$$m_{\text{on}}^{\text{sym}}(u) = \frac{1}{\rho_{\text{on}}^{\text{sym}}(u)} = \frac{1}{\rho_0} \cdot (1 + \exp(-u)) \quad , \tag{34}$$

$$m_{\text{off}}^{\text{sym}}(u) = \frac{1}{\rho_{\text{off}}^{\text{sym}}(u)} = \frac{1}{\rho_0} \cdot (1 + \exp(u)) \quad . \tag{35}$$

Notably, one can identify a single translation factor $F(u)$ between the two systems,

$$\frac{m_{\text{on}}^{\text{sym}}(u)}{m_{\text{on}}(u)} = \frac{\rho_0^{-1} \cdot (1 + \exp(-u))}{\tau \cdot \exp(-u)} \tag{36}$$

$$= \frac{\rho_0^{-1} \cdot (1 + \exp(u))}{\tau} = F(u) \tag{37}$$

$$\frac{m_{\text{off}}^{\text{sym}}(u)}{m_{\text{off}}(u)} = \frac{\rho_0^{-1} \cdot (1 + \exp(u))}{\tau} = F(u) \tag{38}$$

which is given by,

$$F(u) = \underbrace{\frac{1}{\tau \rho_0}}_{\text{const.}} \cdot (1 + \exp(u)) \quad . \tag{39}$$

Note that $F(u)$ is strictly positive and increases monotonically with increasing membrane potential u. Furthermore, note that large values $F(u)$ signify that the spike-based dynamics is fast in comparison with the symmetric dynamics. Hence, for larger u transition times are considerably shortened in the spike-based system. In other words, the asymmetric dynamics of spiking neurons increases specifically the *on*- and *off*-transition rates of those neurons with high membrane potentials u (i.e. neurons with strong input and/or high biases). This makes sense since *off* transitions in the symmetric case can be arbitrarily slowed down for large u, see equation (35), whereas the spike-based system will necessarily fall back to an *off* state on a regular basis regardless of u.

According to equation (M8), high membrane potentials u reflect large energy barriers in the energy landscape. Therefore, the increase of transition rates for large u in the spike-based system (due to deterministic *on→off* transitions) means that large energy barriers are crossed more frequently than in the symmetric system. In particular, *on→off* transitions to high-energy states become considerably more likely due to equation (38). Nevertheless, it should be stressed that on average the spike-based system does not spend more *time* in high-energy states (both systems sample from the same $p(\mathbf{x})$), because according to equation (37) also the transition back to the corresponding *on* state (i.e. the lower energy state) happens at an increased rate for large u. The critical observation is that the return to the identical previous state *can* be intercepted: While the neuron is *off*, other neurons are given the brief opportunity to spike before the previous state is restored, and may thereby, e.g., escape from a previously inhibited state. This is particularly obvious in the context of WTA circuits, where such brief periods of *off*-time of the current winner allow other neurons to take over. Altogether, as we demonstrated in Fig. 2, it is observed that this enhanced utilization of exploratory moves leads to improved search for low energy states in the asymmetric spike-based system, by facilitating fast escape routes from deep local minima which are not available to such extent in a symmetric system.

4.3 ASYMMETRY FACILITATES GOAL-DIRECTED TRANSITIONS

Equation (39) implies that spike-based transition frequency is enhanced in proportion to u. It was already noted above that this encourages exploratory *on→off* transitions which may facilitate the escape from

local minima. But clearly also *off*→*on* transitions are affected by equation (39). In particular, consider a situation where a group of neurons in the *off* state is competing for emitting the next spike (e.g. in a WTA circuit). Those neurons with the highest membrane potentials are particularly eager to fire. Suppose, for example, that there are two neurons with $u_a = 3$ and $u_b = 5$, and all other neurons have considerably lower u. According to equation (37), transition rates in the spike-based system are increased to a greater extent in neurons with higher u: In the symmetrized non-spiking system, transition rates scale with $\sigma(u)$ and are therefore approximately equal for the two neurons a and b (due to saturation of the sigmoid function). In the spike-based system, however, instantaneous transition rates scale with $\exp(u)$ and thus the competition will be much easier to win by the neuron which is most eager to fire (i.e. neuron b in the example). Clearly, this makes a substantial difference in the dynamics and performance of the stochastic search, especially since u_k reflects the drop in energy that can be gained by turning on some neuron k. In particular, it means that a spike-based system is not only more exploratory in the “up-hill” direction (*on*→*off* transitions towards higher energy levels), but also more goal-directed in the “down-hill” direction.

Obviously, the enhanced agility with respect to some transitions must come at a price. Indeed, those transitions which bring about only small changes in the energy landscape (transitions with small) are considerably disadvantaged by the spike-based dynamics. In terms of convergence properties, however, this seems to be a small price to pay, since stochastic search appears in practice more frequently impeded by the presence of large energy barriers. [2]

4.4 COMPARISON OF SOLVING TSP BY A SPIKING NETWORK AND BY A BOLTZMANN MACHINE

Boltzmann machines are artificial (non-spiking) neural networks with stochastic binary units which have biases b_k and where connections between units are required to be symmetric, i.e. $w_{kl} = w_{lk}$, **Hinton and Sejnowski** (1983); **Hinton et al.** (1984). In order to facilitate a fair comparison between spiking network and Boltzmann machines, we adapted the previously described TSP network architecture to include only symmetric weights between neurons. This can be done by replacing di-synaptic inhibitory links mediated through WTA auxiliary neurons by direct inhibitory connections among principal neurons (in violation of Dale’s law). The activation of a principal neuron in a WTA circuit will therefore automatically and directly inhibit all other principal neurons in the same WTA. We note that this adapted architecture has virtually the same energy function as the original implementation with auxiliary inhibitory neurons.

The above described simplification allowed us to perform a fair comparison between spiking network (SN) and Boltzmann machine (BM), since the adapted architecture can be simulated with identical parameters (and leads to an identical stationary distribution $p(\mathbf{x})$) on both systems. The comparison of the number of state changes between SN and BM implementations was done based on 100 runs, each of which was simulated for 100.000 state changes. Both systems were initialized at the beginning of each run in an all-silent state (i.e. all $z_k = 0$). In each run and after every state change we evaluated the current network state, and checked how many problem variables were properly defined. Combining all runs in each case, we calculated how often transitions occurred in each sampler to states with different numbers $N_{undef} \in \{0, \ldots, N'\}$ of undefined problem variables. Based on this information we constructed corresponding histograms for SN and BM. To highlight the differences between the two implementations, we calculated the ratios between the normalized histogram values for SN and BM (Fig. 2C). For the convergence speed comparison, in each run we calculated after each state change the cumulative minimum and mean performance during the whole time leading up to that state change. This was first done for each of the 100 network runs individually. The results for each number of state changes were then averaged over all runs.

[2] In principle, one sees from equation (39) that on the other hand transitions with negative u are disadvantaged by the spike-based dynamics. In the context of this paper negative u only occur in neurons which are currently inhibited in a WTA circuit.

4.5 FURTHER DETAILS TO THE OBSERVED SPIKE-BASED ENERGY JUMPS IN FIG. 3C

The histograms in Fig. 3C display a striking symmetricity of state transitions with positive and negative energy differences. For the Boltzmann machine this is an expected consequence of detailed balance in Gibbs sampling **Brooks et al.** (2010), which ensures that on average transitions between any two states are observed equally often in either direction. For the spiking network, however, the observed symmetricity cannot be predicted from theoretical considerations alone. This is because detailed balanced does not hold in a strict sense in the spiking network when off-transitions follow a deterministic decay law **Buesing et al.** (2011). Nevertheless, the spiking network dynamics can be easily morphed into a system that obeys detailed balance: When off-transitions are made stochastic with constant rate τ^{-1} (such that on average neurons are on for τ time units before turning off as in the spiking network), the dynamics of the system can be described in terms of a simple Master equation,

$$\frac{dp_t(\mathbf{x})}{dt} = \tau^{-1}\sum_k \left[p_t(\neg x_k, \mathbf{x}_{\backslash k}) \cdot \exp(x_k u_k) - p_t(\mathbf{x}) \cdot \exp((1-x_k)u_k)\right] \tag{40}$$

$$= \tau^{-1} p_t(\mathbf{x}) \sum_k \exp(x_k u_k) \left[\frac{p_t(\neg x_k|\mathbf{x}_{\backslash k})}{p_t(x_k|\mathbf{x}_{\backslash k})} - \exp((1-2x_k)u_k)\right] \tag{41}$$

$$= \tau^{-1} p_t(\mathbf{x}) \sum_k \exp(x_k u_k) \left[\left(\frac{p_t(\neg x_k|\mathbf{x}_{\backslash k})}{p_t(x_k|\mathbf{x}_{\backslash k})}\right)^{1-2x_k} - \exp(u_k)^{1-2x_k}\right] \tag{42}$$

where $p_t(\mathbf{x})$ denotes the probability that the system is in state $\mathbf{x}$ at time t, and $p_t(\neg x_k, \mathbf{x}_{\backslash k})$ denotes the probability of state $\mathbf{x}$ with negated element k. Detailed balance requires that all summands (i.e. the probability flows for all neurons k) are zero at equilibrium, for all possible states $\mathbf{x}$. It is easy to verify that this holds if the NCC given by equation (M5) is fulfilled. Hence, although the spiking network is not strictly in detailed balance, a slightly modified dynamics of the spiking network is. This provides a potential explanation for the symmetricity of the top histogram in Fig. 3C.

Another peculiarity of the top histogram in Fig. 3C (energy jumps in the spiking network) is its highly non-Gaussian shape (the distribution of positive jumps is even bi-modal). It should be stressed that the particular shape of the histogram, including the dip around 15, does not reveal general properties of the spiking network dynamics, but reflects the statistics of the problem instance. Concretely, the fact that positive jumps are bi-modally distributed arises from the architecture of the TSP network (Fig. 2A): Each neuron has at most two neighbors that provide positive input. When only one neighbor is active, the membrane potential is typically between 10 and 14, and when both are active, the membrane potential is typically between 20 and 28. The membrane potential u of a neuron encodes the energy difference of an off-transition, and $-u$ represents the energy difference of an on-transition. Hence, the observed bi-modally distributed energy jumps in the spiking network are a direct consequence of the specific problem architecture. In the Boltzmann machine this effect is not visible since transitions in the relevant range of energy differences are suppressed.

5 DETAILS TO PRINCIPLE 4: INTERNAL TEMPERATURE CONTROL

In order to realize an internal temperature control mechanism for regulating the temperature T of the network energy function according to $E_T(\mathbf{x}) = E(\mathbf{x})/T$ in an autonomous fashion, at least three functional components are required (Fig. 1D): 1. Internally generated feedback signals from circuit motifs reporting on the quality and performance of the current tentative network solution. 2. A *temperature control* unit which integrates feedback signals and decides on an appropriate temperature T. 3. An implementation of the requested temperature change in each circuit motif.

Both circuits motifs, WTA and OR, can be equipped quite easily with the ability to generate internal feedback signals. The WTA condition in a WTA circuit is met if exactly one principal neuron is active in the circuit. If the WTA circuit has strong inhibition then network states with two or more active neurons are very unlikely and can be ignored in practice. Thus, a WTA feedback signal can be generated by simply checking whether *any* principal neuron is active. Concretely, the output of the auxiliary WTA neuron can be used as a feedback signal, since the auxiliary neuron is only activated when one of the principal neurons has fired. Alternatively, for the same reasons the WTA feedback signal may be constructed from the summed outputs of all principal neurons in a WTA circuit.

For the OR circuit motif, the OR condition is met if at least one principal neuron is active. The most straightforward way of implementing an OR feedback signal is therefore to add a feedback neuron which fires as long as one of the principal neurons is active, and remains silent otherwise. This can be achieved in a straightforward manner by a feedback neuron with low bias and excitatory connections from all involved principal neurons. In simulations a slightly different implementation has proved even more effective, which can be used when all principal neurons involved in the OR circuit are also part of some WTA circuit. Then, a negative feedback signal (one which is active when the OR condition is violated) can be generated by adding an auxiliary neuron with low bias which receives connections from all other neurons in the WTA circuits of the involved principal neurons. The rationale behind this implementation of an OR feedback signal is as follows: Whenever some principal neuron k, which is involved in the OR circuit and in addition in some WTA circuit, is not active, most of the time some other neuron in the WTA circuit of neuron k must be active. Hence, whenever the OR constraint is violated and all K principal neurons involved in the OR circuit are inactive, the feedback neuron will see that in each of the involved WTA circuits some other neuron is active. A more detailed description of how this was implemented as part of the temperature control mechanism for 3-SAT problems is given in Section ”Details to 3-SAT application“.

Regarding the temperature control unit, one can think of various smart strategies to integrate feedback signals in order to decide on a new temperature. In the simplest case, a temperature control unit has two temperatures to choose from: one for exploration (high temperature), and for stabilization of good solutions (low temperature). A straightforward way of selecting a temperature is to remain at a moderate to high temperature (exploration) by default, but switch temporarily to low temperature (stabilization) whenever the number of positive feedback signals exceeds some threshold, indicating that almost all (or all) constraints in the circuit are currently fulfilled.

Concretely, such internal temperature control unit can be implemented via a temperature control neuron with a low bias and connection strengths from feedback neurons in each circuit in such a manner that the neuron’s firing probability reaches non-negligible values only when all (or almost all) feedback signals are active. When circuits send either positive or negative feedback signals, the connection strengths from negative feedback neurons should be negative and can be chosen in such a manner that non-negligible firing rates are achieved only if all positive feedback but none of the negative feedback signals are active. Whenever such temperature control neuron is active it indicates that the circuit should be switched to the low temperature (stabilization) regime.

REFERENCES

Berkes, P., Orban, G., Lengyel, M., and Fiser, J. (2011), Spontaneous cortical activity reveals hallmarks of an optimal internal model of the environment, *Science*, 331, 83–87

Bishop, C. M. (2006), Pattern Recognition and Machine Learning (Springer, New York)

Brooks, S., Gelman, A., Jones, G., and Meng, X.-L. (2010), Handbook of Markov Chain Monte Carlo: Methods and Applications (Chapman & Hall)

Buesing, L., Bill, J., Nessler, B., and Maass, W. (2011), Neural dynamics as sampling: A model for stochastic computation in recurrent networks of spiking neurons, *PLoS Computational Biology*, 7, 11, e1002211, doi:10.1371/journal.pcbi.1002211

Habenschuss, S., Jonke, Z., and Maass, W. (2013), Stochastic computations in cortical microcircuit models, *PLoS Computational Biology*, 9, 11, e1003311, doi:10.1371/journal.pcbi.1003311

Hinton, G. E. and Sejnowski, T. J. (1983), Optimal perceptual inference, in Proceedings of the IEEE conference on Computer Vision and Pattern Recognition, 448–453

Hinton, G. E., Sejnowski, T. J., and Ackley, D. H. (1984), Boltzmann machines: constraint satisfaction networks that learn, Technical Report CMS-CS-84-119, CMU Computer Science Department

Jolivet, R., Rauch, A., Lüscher, H., and Gerstner, W. (2006), Predicting spike timing of neocortical pyramidal neurons by simple threshold models, *Journal of Computational Neuroscience*, 21, 35–49

Pecevski, D., Buesing, L., and Maass, W. (2011), Probabilistic inference in general graphical models through sampling in stochastic networks of spiking neurons, *PLoS Computational Biology*, 7, 12, e1002294, doi:10.1371/journal.pcbi.1002294

Plischke, M. and Bergersen, B. (2006), Equilibrium Statistical Physics (World Scientific)

Schneidman, E., Berry, M., Segev, R., and Bialek, W. (2006), Weak pairwise correlations imply strongly correlated network states in a neural population, *Nature*, 440, 7087, 1007–1012

Truccolo, W., Eden, U., Fellows, M., Donoghue, J., and Brown, E. (2005), A point process framework for relating neural spiking activity to spiking history, neural ensemble, and extrinsic covariate effects, *Journal of Neurophysiology*, 93, 2, 1074–1089

Yamanaka, K., Agu, M., and Miyajima, T. (1997), A continuous-time asynchronous Boltzmann machine, *Neural Networks*, 10, 6, 1103–1107
